# Association of Screen Time With Physical and Mental Health in University Students: Systematic Review and Meta-Analysis

**DOI:** 10.2196/93848

**Published:** 2026-10-26

**Authors:** Gongbinxuan Wu, Claire Eades, Louise Hoyle

**Affiliations:** 1 Faculty of Health Sciences, University of Stirling Stirling, Scotland United Kingdom

**Keywords:** anxiety, body problem, depression disorder, dry eye syndromes, psychological stress, screen time, sleep, university student, well-being

## Abstract

**Background:**

Screen use is almost universal, and numerous studies have highlighted both the benefits and the risks of this digital integration. However, evidence on the association between screen time and health outcomes among university students remains inconsistent. Substantial methodological variation has limited the synthesis and interpretation of existing findings.

**Objective:**

This study aimed to (1) quantify the associations between screen time and health outcomes as primary analysis; (2) quantify the association between screen time and physical (sleep quality, eye health, weight-related) and mental health outcomes (depression, psychological distress/stress, combined mental health, and well-being); and (3) identify study-level characteristics that explain between-study heterogeneity by using subgroup and meta-regression analyses.

**Methods:**

PubMed, PsycINFO, CINAHL, and Web of Science were searched, with final searches conducted on July 11, 2026, and reference lists were additionally screened. Eligible studies included university students aged 17 years or older, assessed screen time as duration, and reported physical or mental health outcomes with sufficient statistical data for effect-size calculation. We followed the PRISMA (Preferred Reporting Items for Systematic Reviews and Meta-Analyses) checklist. Data were synthesized in RStudio using the restricted maximum likelihood estimator and Hartung-Knapp-Sidik-Jonkman adjustments. Subgroup analyses and meta-regressions was examined. Small-study effects, leave-one-out, and influence diagnostics were also conducted.

**Results:**

A total of 82 studies (83 independent samples; 161,340 participants) were included. Longer screen time duration was significantly associated with poorer health outcomes overall (k=83; *r*=0.175, 95% CI 0.133-0.217; *P*<.001; 95% prediction interval –0.184 to 0.493), with substantial between-study heterogeneity (*I*^2^=98.35%; Q_82_=4744.45; *P*<.001). Pooled associations were similar for mental health (k=38; *r*=0.176, 95% CI 0.124-0.227) and physical health outcomes (k=45; *r*=0.177, 95% CI 0.108-0.243) and broad health domain did not significantly moderate the association (*P*=.96). Within mental health, outcome type significantly moderated the association (*P*=.03). The association was the strongest for depression (k=21; *r*=0.230, 95% CI 0.167-0.291) and was weaker for well-being (k=7; *r*=0.040, 95% CI –0.146 to 0.223). Combined mental health outcomes were also significant (k=8; *r*=0.170, 95% CI 0.076-0.261), whereas stress was not (k=2; *r*=0.103, 95% CI –0.222 to 0.407). Within physical health, screen time was significantly associated with poorer sleep quality (k=26; *r*=0.120, 95% CI 0.083-0.158) and eye health outcomes (k=16; *r*=0.277, 95% CI 0.089-0.446), but not weight-related outcomes (k=3; *r*=0.071, 95% CI –0.222 to 0.351); differences across physical outcome types were not significant. Study-level moderators did not meaningfully explain the remaining heterogeneity.

**Conclusions:**

Greater screen time was associated with poorer health outcomes among university students, but the magnitude of association varied across specific outcomes and substantial unexplained heterogeneity remained. The predominance of cross-sectional designs, self-reported screen time, and sparse evidence for some outcomes limits causal inference and precision. Future research should prioritize longitudinal designs and standardized measurement approaches.

**Trial Registration:**

PROSPERO CRD42024539034; https://www.crd.york.ac.uk/PROSPERO/view/CRD42024539034

## Introduction

Digital technologies have become deeply embedded in modern society [1]. Screen-based devices such as smartphones, tablets, computers, and televisions now shape how people interact, behave, and live their daily lives. Numerous studies have highlighted both the benefits and the risks of this digital integration [2]. Excessive screen exposure has been associated with adverse physical health outcomes such as obesity, sedentary behavior, and sleep disturbances [3,4]. Furthermore, accumulating evidence indicates that prolonged screen exposure is associated with poorer mental health, including increased symptoms of depression, anxiety, and low self-esteem, as well as strained interpersonal relationships [5,6].

University students rely heavily on screens not only for entertainment and social interaction but also for academic learning, online assessment, communication with peers and instructors, employment, and administrative activities [7-9]. Evidence indicates that students report an average of 8.3 hours of daily screen time [10], which rises by 2 to 3 hours when objectively measured with accelerometers [11,12]. Their usage levels are comparable to, or exceed, those of other young adult groups [9,13,14]. At the same time, university life is also a critical developmental stage marked by major transitions, including relocation, academic stress, social pressures, and lifestyle changes [15-17]. Therefore, understanding the health implications of screen time among university students is essential for informing evidence-based health promotion strategies within universities.

Growing evidence suggests that prolonged screen exposure may adversely affect multiple dimensions of health. A systematic review of students and adolescents reported that social media addiction was significantly associated with anxiety, depression, fear of missing out, loneliness, and lower self-esteem, highlighting the broad psychological consequences of excessive digital engagement [18]. More recently, another systematic review synthesizing evidence on screen exposure across university populations concluded that prolonged screen use was associated with a range of adverse outcomes, including poorer sleep quality, musculoskeletal discomfort, digital eye strain, reduced physical activity, and impaired academic functioning [19]. Despite these important advances, several important evidence gaps remain. First, previous reviews have primarily focused on quantifying overall associations between screen exposure and health outcomes and have examined potential sources of heterogeneity, whereas evidence regarding whether associations vary systematically according to study quality, geographic region, or screen exposure assessment characteristics remains limited and inconsistent. Second, although a wide range of physical and mental health outcomes has been examined, evidence has generally been synthesized separately or descriptively, with limited direct comparisons of the consistency and magnitude of associations across different health domains. Finally, several large observational studies have been published in recent years, making an updated quantitative synthesis necessary to provide a more comprehensive and contemporary assessment of the available evidence.

Therefore, this systematic review and meta-analysis was conducted to provide a more comprehensive synthesis of the current evidence regarding screen time and health outcomes among university students aged 17 years and older. Specifically, this review aimed to (1) quantify the associations between screen time and health outcomes as primary analysis; (2) separately quantify the association between screen time and physical (sleep quality, eye health, weight-related outcomes) and mental health outcomes (depression, psychological distress/stress, combined mental health, and well-being); and (3) identify study-level characteristics that explain between-study heterogeneity using subgroup and meta-regression analyses.

## Methods

### Study Protocol and Registration

This review was conducted in compliance with PRISMA (Preferred Reporting Items for Systematic Reviews and Meta-Analyses) [20] guidelines to ensure a rigorous and transparent evaluation of the review process. For details of items included in the PRISMA checklist, please see Multimedia Appendix 1. The protocol was registered in the PROSPERO (International Prospective Register of Systematic Reviews) database (registration number CRD42024539034). Several amendments were made to the registered protocol. The geographic restriction to Western countries was removed, CINAHL was added to the database search, classification of screen-time exposures according to categories reported in the included studies rather than the planned active-passive distinction, replacement of the Joanna Briggs Institute appraisal tool with the AHRQ (Agency for Healthcare Research and Quality) methodology checklist, and refinement of the synthesis methods to include moderators. Additional moderator and sensitivity analyses and GRADE (Grading of Recommendations Assessment, Development, and Evaluation) assessment of the certainty of evidence were also incorporated.

### Information Sources and Search Strategy

The search strategies were developed by 2 researchers with expertise in systematic reviews and peer-reviewed by a librarian. The following electronic databases were searched using a comprehensive search strategy to identify relevant studies: PubMed (via National Center for Biotechnology Information), PsycINFO (via EBSCOhost), and CINAHL (via EBSCOhost) up to December 31, 2024. Databases were searched individually rather than simultaneously on a single multidatabase platform. No study registries were searched. The search strategies combined terms representing 3 core concepts: the target population (university students), screen-based exposures, and relevant mental and physical health outcomes. The search strategies used a combination of free-text search terms and database-specific controlled vocabulary, including MeSH (Multimedia Appendix 2). Synonymous terms were combined with the Boolean “OR” and then combined with the Boolean “AND.” In all databases, truncation symbols were used in text word searches to capture variations in phrasing and spelling. No published search filters were used. Search strategies from previous literature reviews were not adapted or reused. The literature search was updated on July 11, 2026, using search strategies adapted from the original searches, with Web of Science Core Collection (via Clarivate) additionally searched during the update. Database-specific limits were applied and are fully reported in Multimedia Appendix 2. Owing to differences in database functionality, the precision of the available date limits varied across databases: PubMed permitted restriction to the day level, APA PsycINFO and CINAHL Ultimate to the month level, and Web of Science Core Collection to the year level. The reference lists of all included studies and relevant review studies were manually screened to identify any additional eligible studies not captured by the database search. Records retrieved from each database were exported and imported into EndNote X9.3.3 (Build 15659; Clarivate). Duplicate studies were first removed using EndNote X9, and any remaining duplicates were removed manually. No restrictions were placed on publication date in the original search, whereas the updated searches of PubMed, PsycINFO, and CINAHL were restricted to records published since the completion of the original search. Only English-language and peer-reviewed studies were considered eligible for inclusion. No additional online or print sources were purposefully searched or browsed. No additional studies or unpublished data were sought by contacting authors. The search process and its reporting followed the PRISMA-S (Preferred Reporting Items for Systematic Reviews and Meta-Analyses literature search extension; Multimedia Appendix 3) guidelines to ensure transparency, completeness, and reproducibility of each search component [21].

### Eligibility Criteria

Eligibility criteria were defined a priori and are summarized in Multimedia Appendix 4. Briefly, studies were eligible if they included university students aged 17 years or older, assessed screen time duration as the exposure of interest, and reported at least 1 predefined physical or mental health outcome. Screen time could be measured using objective methods (eg, device-based tracking or accelerometry) or self-reported questionnaires. Eligible study designs included observational studies (cross-sectional, cohort, and case-control studies) and intervention studies for which baseline associations between screen time and health outcomes could be extracted. Only peer-reviewed studies published in English were included. Predefined minimum sample size thresholds were applied, consistent with previous evidence syntheses [22]. Observational studies were required to include at least 300 participants, whereas randomized and nonrandomized intervention studies were required to include at least 30 participants in the intervention group. Studies focusing on clinical drug trials or specific screen addiction constructs (eg, smartphone or gaming addiction) rather than screen time duration were excluded.

### Study Selection

Study screening was conducted using the Rayyan web-based tool [23]. No automation tools were used in this process. Three researchers (GW, CE, and LH) independently screened all titles and abstracts against the inclusion criteria. The first reviewer (GW) reviewed all the results from the databases, and the second review was split between 2 other authors (CE and LH). For any studies where it was unclear if the inclusion criteria were met, the study was carried forward into the full-text review. Disagreements between reviewers were resolved through discussion, and final decisions regarding study inclusion were made by consensus among all 3 researchers.

### Data Extraction

Data extraction forms were created for this review and piloted prior to full data extraction. Data were extracted by 1 reviewer (GW) using a standardized Microsoft Excel spreadsheet. Any uncertainties were discussed with a second reviewer (CE). The following information was extracted from each eligible study: first author, country, year of publication, study design, sample characteristics and study objectives, screen time definition and screen time measurement tools, health indicators and outcomes. Where multiple statistical models were reported, effect estimates from the most fully adjusted models were extracted whenever available. When multiple eligible health outcomes or independent samples were reported within a study, data were extracted separately for each outcome or sample. Studies were excluded from the quantitative synthesis when an eligible effect size could not be derived from the article or supplementary materials. Corresponding authors were not systematically contacted, and missing effect size data were not imputed.

For each study, effect sizes and relevant methodological characteristics were recorded. Potential moderating variables to account for between-study heterogeneity were extracted. Studies were classified according to the primary screen time exposure evaluated in the original study. Exposure categories included total screen time, total social media screen time, recreational screen time, smartphone screen time, and computer screen time. Total screen time referred to measures assessing overall or aggregated screen exposure across devices or activities, whereas the remaining categories reflected more specific device, activity, or purpose-based screen time measures. Prespecified moderators included the percentage of female participants, screen time exposure category, health outcome category, study design (cross-sectional or longitudinal study), year of publication, and geographic location (Asia, Europe, Middle East, North America, South America, or Others).

Health outcomes were classified into mental and physical health domains based on the constructs assessed in the original studies. Mental health outcomes were further categorized as depression, stress, well-being, or combined mental health outcomes. The combined mental health category comprised estimates representing at least 2 distinct mental health constructs (eg, anxiety, depression, or stress). These estimates were either based on a multidimensional or composite measure reported in the original study or obtained by averaging eligible effect sizes for different mental health constructs within the same independent sample, following the procedures described in the “Data Synthesis” section. Physical health outcomes were categorized as eye health, sleep quality, and weight-related outcomes (study characteristics, specific health outcome measures or instruments, and corresponding outcome categories are reported in Table S3 in Multimedia Appendix 5, and the definitions and measurement instruments used for eye health outcomes were provided in Table S4).

### Study Quality Assessment

Each study was evaluated by 2 independent coders (GW and CE) for quality based on the adopted 11-item AHRQ methodology checklist, with each item coded as 0 (“No”/“Unclear”/“Not Applicable”) or 1 (“Yes”) [24] (Table S5 in Multimedia Appendix 6). This approach was adopted because the majority of included studies were observational in design and reported heterogeneous outcome measures, making study-level assessment the most consistent and feasible strategy. The appraisal focused on key domains affecting internal validity, including participant selection, measurement of exposures and outcomes, and control of confounding factors. Intercoder agreement for the overall quality score was good (intraclass correlation coefficient=0.75). Discrepancies were resolved via the third independent coder (LH). Studies scoring less than 4 were classified as “low quality,” 4-7 as “moderate quality,” and 8-11 as ”high quality” [25] (Table S6 in Multimedia Appendix 6).

### Certainty of Evidence

Following the methodological quality assessment, the overall certainty of evidence for each health outcome was evaluated using the GRADE approach [26]. As the included studies were predominantly observational in design, the certainty of evidence was initially rated as low and was then assessed according to 5 domains: risk of bias, inconsistency, indirectness, imprecision, and publication bias [26,27]. Potential upgrading factors, including a large magnitude of effect, a dose-response gradient, and plausible residual confounding, were also considered where applicable [28]. Risk of bias was informed by the 11-item AHRQ methodological quality assessment, considering the methodological limitations of studies contributing to each outcome. Inconsistency was assessed using *I*^2^, τ^2^, Cochran Q, and the variability of effect estimates [29]; indirectness was assessed according to the alignment of populations, exposures, and outcomes with the review question [30]; and imprecision was evaluated using the 95% CIs and the amount of available evidence [31]. Evidence was downgraded by 1 or 2 levels for serious or very serious concerns, respectively. Finally, the certainty of evidence was categorized as high, moderate, low, or very low [26]. The GRADE assessment was performed independently by 2 reviewers (GW and CE), with disagreements resolved through discussion with a third reviewer (LH).

### Data Synthesis

When multiple studies reported analyses based on the same underlying sample, the study with the largest sample size and most comprehensive information was selected. Adjusted statistics were selected over unadjusted statistics whenever available. To maintain statistical independence, each independent sample ultimately contributed one effect size to the primary meta-analysis.

When multiple eligible measures of the same exposure or multiple eligible health outcomes, including outcomes across mental and physical health domains, were reported within a study, more global measures of screen time (eg, total screen time) and more comprehensive measures of the relevant health outcome were prioritized over device-specific measures or individual symptom items, respectively. Similar to other meta-analyses [32-35], when a single study measured screen time and/or physical and mental health problems at multiple time points, effect sizes with the largest temporal distance between measures were selected. When a single effect size could not be selected based on these criteria, multiple eligible effect sizes within the same health domain were averaged to derive a single effect estimate for that independent sample. When an independent sample reported eligible outcomes in both the mental and physical health domains, the domain-specific effect estimates obtained using the procedures above were compared, and the estimate with larger absolute magnitude was retained for the primary meta-analysis, with its original sign preserved. When Pearson correlation coefficients (*r*) were unavailable, standardized *β* coefficients with values ranging between –0.50 and 0.50 were considered as approximations of *r*, in accordance with the method described in Peterson and Brown [36].

### Statistical Analysis

Effect sizes reported in the primary studies were imported into Comprehensive Meta-Analysis (CMA, version 3.7 Biostat) [37]. Where necessary, reported effect sizes were converted into a common metric of Pearson correlation coefficient (*r*) using the software’s built-in effect size conversion procedures. Correlation coefficients were subsequently transformed to Fisher *z* values to stabilize sampling variances and improve the normality of their sampling distributions [38]. The corresponding SEs of Fisher *z* values were calculated within CMA, and the resulting dataset was exported for subsequent analyses in R (version 2024.09.0+375; R Foundation for Statistical Computing).

All meta-analyses were conducted in R using the *metafor* package [39]. CMA was used solely for effect size conversion and standardization, whereas all pooled analyses and statistical inference were performed in RStudio. Random-effects models were applied throughout because substantial methodological and clinical heterogeneity was anticipated across the included studies, including differences in participant characteristics, screen time exposure type and format, outcome measures, study design, and study conduct geography. Between-study variance (τ^2^) was estimated using the restricted maximum likelihood (REML) estimator [39]. Statistical inference for pooled effect estimates was based on the Hartung- Knapp-Sidik-Jonkman (HKSJ) adjustment, which generally provides improved CI coverage compared with conventional random-effects inference, particularly when the number of studies is small [40].

Pooled Fisher *z* estimates and corresponding 95% CIs were back-transformed into Pearson correlation coefficients (*r*) to facilitate interpretation [41]. Correlation coefficients were interpreted using empirically derived benchmarks proposed by Gignac and Szodorai [42], which were subsequently supported and discussed by Funder and Ozer [43], whereby values of approximately 0.10, 0.20, and 0.30 represent relatively small, typical, and relatively large effects, respectively. These benchmarks were developed from the empirical distribution of more than 700 meta-analytically derived correlations and have been recommended for the interpretation of correlation coefficients in individual differences research [42]. Forest plots were used to visually display individual study effect estimates and pooled estimates with 95% CIs.

Between-study heterogeneity was assessed using Cochran Q statistic, *I*^2^, and τ^2^ [39]. In addition to pooled effect estimates and 95% CIs, 95% prediction intervals were calculated for the primary analyses to estimate the range within which the true effect of a future comparable study would be expected to lie [44]. Potential sources of heterogeneity were explored across the overall dataset using univariable random-effects meta-regression analyses. Subgroup analyses were conducted for categorical moderators, whereas meta-regression was performed for continuous moderators using REML estimation with HKSJ inference. Differences between subgroups were evaluated using tests of between-group heterogeneity. Potential small-study effects were assessed through visual inspection of funnel plots and the Egger test [45,46], supplemented by Duval and Tweedie’s [47] trim-and-fill procedure. A 2-tailed *P* value of .05 was used for primary analyses, whereas a more conservative significance threshold of *P*<.01 was applied to moderator analyses because multiple moderators were examined [48]. Sensitivity analyses were conducted to assess the robustness of the findings. These included Leave-one-out analysis to investigate the influence of a single study on the pooled effect [44], influence diagnostics to identify potentially influential studies [49], and reanalysis after excluding studies classified as low quality. An additional post hoc sensitivity analysis was conducted for the eye-health outcome by excluding studies that did not use an established, validated eye-health instrument in its standard form, to assess the robustness of the pooled estimate to differences in outcome measurement [50].

### Moderator Selection and Analysis

Potential moderators were selected based on their conceptual, contextual, or methodological relevance to between-study heterogeneity rather than on data availability alone. These exploratory moderator analyses were conducted across the overall dataset. Screen-time exposure type represented differences in the nature of the exposure. Geographic region, sex composition, and mean participant age represented contextual and population characteristics; and study design, study quality, and year of publication represented potential methodological or temporal sources of heterogeneity. Study quality was entered into meta-regression as the continuous AHRQ score rather than as the categorical quality classification. Each moderator was examined separately using random effects meta-regression to limit model complexity and reduce the risk of overfitting [51]. Studies with missing information for a specific moderator were excluded only from the corresponding meta-regression and remained eligible for analyses of other moderators.

## Results

### Results of Study Selection

A total of 25,600 records were identified through database searching, including PubMed (n=12,735), APA PsycInfo (n=7276), CINAHL Ultimate (n=4208), and Web of Science Core Collection (n=1381). After removing 7683 duplicates, 17,917 remained for title and abstract screening. Among them, 16,767 records were excluded because they did not meet the eligibility criteria. The remaining 1150 records were included in the full-text screening, and 23 studies that were inaccessible or retracted were first excluded. Next, we excluded studies that focused on screen use addiction (n=702), studies with participants aged less than 17 years old (n=108), studies without relevant measures of screen time or physical or mental health (n=55), studies without usable statistics (n=77), cross-sectional studies with sample size less than 300 (n=83), and studies not published in English (n=26). Consequently, 76 studies were identified through the database searches. An additional 6 eligible studies were identified through citation searching of previous systematic reviews and other online sources. Ultimately, 82 studies [17,52-132] (with 83 unique samples) were included in the meta-analysis. The study selection process is presented in Figure 1. Studies that appeared to meet the eligibility criteria but were excluded after full-text assessment, together with the reasons for their exclusion, are presented in Multimedia Appendix 4.

**Figure 1:**
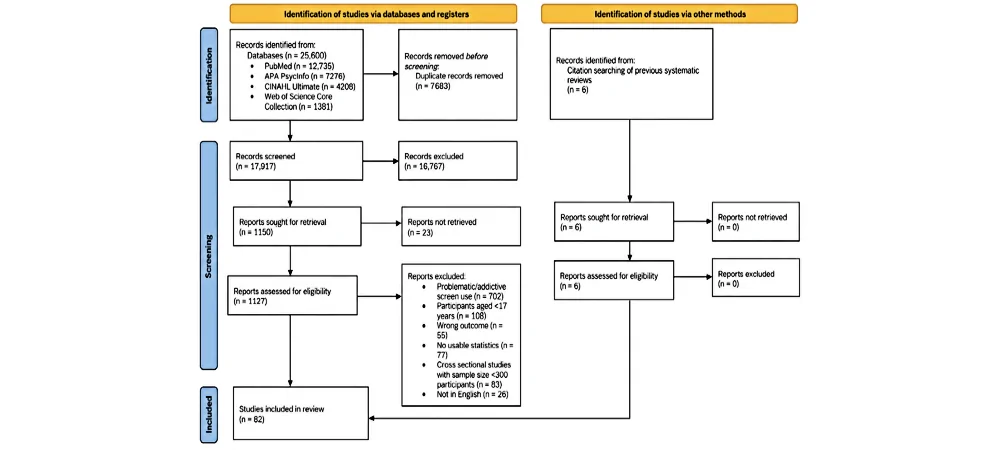
PRISMA (Preferred Reporting Items for Systematic Reviews and Meta-Analysis) 2020 flow diagram of the study selection process.

### Characteristics of Included Studies

The 82 included studies [17,52-132] comprising 83 independent samples were published between 2008 and 2026. One study reported 2 independent samples, which were treated as separate analytic samples because they comprised distinct participant groups with no participant overlap [82]. Of these, 75 (91.5%) studies [52,54-88,90-103,105-112,114-119,122-132] were published from 2020 onward, indicating a substantial increase in research on screen time and health among university students in recent years. The 83 included samples comprised 161,340 participants, of whom 57.2% (92,286/161,340) were female individuals. Sample sizes ranged from 321 to 49,051 participants. The mean age was not reported in 22 samples [52,59-61,66,68,69,78,79,90,103,107-109,113,116,117,119,122,126, 131,132]. Geographically, 49 (59%) samples were conducted in Asia [17,53,57,65,68,75,76,78,79,82,84-93,95,97-105,107, 109,110,114,116-125,128-132], followed by 11 (13.3%) samples in North America [69,71,72,74,77,102,108,112,113,115,127], 11 (13.3%) were from other region samples [52,56, 58-61,63,66,67,70,126], 8 (9.6%) were from Europe [54,55,62,64,80,83,96,106], and 4 (4.8%) were from South America [73,81,94,111]. Regarding study design, 5 studies used a longitudinal design [71,118,120,125,128], whereas the remaining studies were cross-sectional studies. Regarding screen time exposure, 45 studies assessed total screen time [17,52,54,56-60,63,66,70,73-75,77,78,80-83,86,88,89,93,94,96-100, 102,105-107,110,112,114-116,119,121,124,126,127,129], 14 social media screen time [62,68,69,71,84,90,91,95,103,104, 109,111,118,130], 13 smartphone screen time [53,61,64,65, 67,76,79,85,92,101,108,117,132], 7 recreational screen time [55,72,113,120,123,128,131], and 3 computer screen time [87,122,125]. Among the 83 samples, 38 samples from 37 studies reported on mental health outcomes [17,52,57,68,71, 72,74,75,77,79,82,86,91,94,95,97-99,102,104,108-111,116,117,119-125, 129-132], while 45 samples reported physical health outcomes [53-56,58-67,69,70,73,76,78,80,81,83-85,87-90,92,93,96,100,101,103, 105-107,112-115,118,126-128]. None of the included studies examined the association between screen time and body composition or metabolic syndrome. None of the included studies measured screen time exposure by objective measurement. The characteristics of the included studies are summarized in Multimedia Appendix 5.

### Methodological Quality of Included Studies

In this study, the methodological quality of the included studies was predominantly moderate. Among the 82 included studies, 16 (19.5%) were classified as high quality [17,56,67,72, 75,83,88,94,98,111,117,118,120,128,130,132], 60 (73.2%) as moderate quality [52,54,55,57-66,68-71,73,74,76-81,84,85,87, 90-93,95-97,99,101-105,107-110,112-116, 121-127,129,131], and 6 (7.3%) as low quality [53,82,86,89,100,106]. Most studies clearly reported the study design, data sources, and study period. However, methodological limitations were commonly observed in the reporting of blinding procedures, control of confounding factors, and handling of missing data (Table S6 in Multimedia Appendix 6). These findings indicate variability in methodological rigor across the included studies.

### Certainty of Evidence

The certainty of evidence for all 7 health outcomes was assessed using the GRADE approach. Because the evidence was derived from observational studies, certainty was initially rated as low and was subsequently downgraded according to the applicable GRADE domains. The full GRADE evidence profile and summary of findings were provided in Multimedia Appendix 7. The certainty of evidence was very low for all 7 outcomes. Certainty was downgraded for inconsistency for depression, combined mental health, well-being, sleep quality, eye health, and weight-related outcomes. Risk-of-bias concerns resulted in downgrading for combined mental health, stress, eye health, and weight-related outcomes. Imprecision resulted in further downgrading for well-being, stress, eye health, and weight-related outcomes, while serious indirectness was only for combined mental health outcomes because of variation in the constructs included in the composite measures.

### Synthesis of Findings

#### Association Between Screen Time and Health Outcomes

Across 83 independent samples from 82 studies (Figure 2 [17,52-132]), the random-effects meta-analysis using REML estimation with the HKSJ adjustment showed a significant pooled correlation between screen time and health outcomes (*r*=0.175, 95% CI 0.133-0.217; *P*<.001). Considerable heterogeneity was observed across the included samples (τ^2^=0.033, *I*^2^=98.35%; Q_82_=4744.45; *P*<.001). The 95% prediction interval ranged from –0.184 to 0.493.

**Figure 2:**
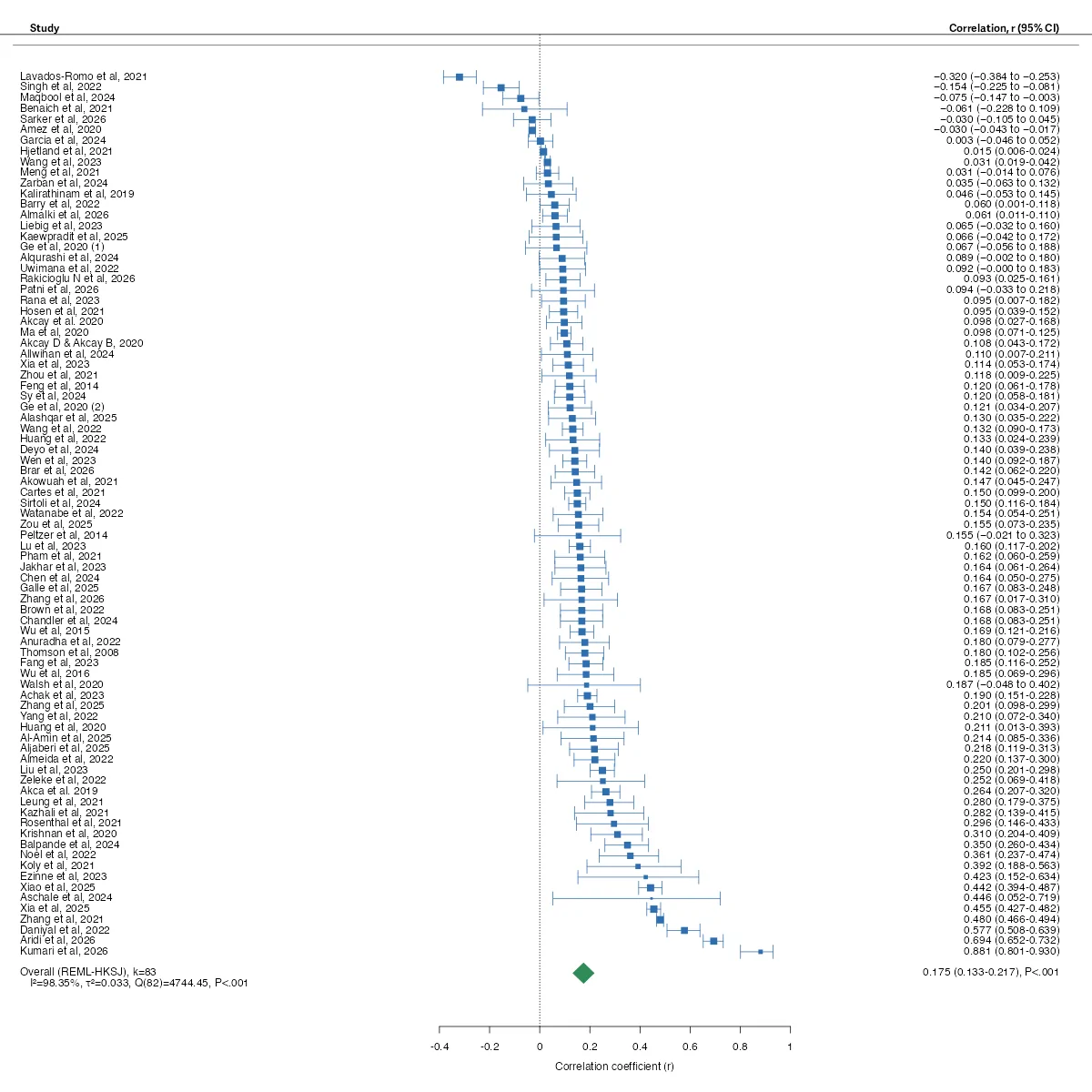
Forest plot of the association between screen time and health outcomes among university students. HKSJ: Hartung-Knapp-Sidik-Jonkman; REML: restricted maximum likelihood.

#### Assessment of Small Study Effects

In this meta-analysis and systematic review, small-study effects were assessed through visual inspection of the funnel plot and Egger test. Funnel plot of the association between screen time and health outcomes in Figure S1 in Multimedia Appendix 8. The vertical line represents the pooled effect estimate from the random-effects models. Visual inspection suggested funnel plot asymmetry. Egger test indicated significant small-study effects (t_81_=3.34; *P*=.001). Duval and Tweedie’s [47] trim-and-fill analysis did not impute any potentially missing studies (k=0). Therefore, the pooled estimate was unchanged.

#### Sensitivity Analysis

Leave-one-out analyses showed that the pooled effect remained statistically significant after the exclusion of any single effect size, with pooled correlation coefficients ranging from 0.165 to 0.181 (Figure S2 in Multimedia Appendix 9). Influence diagnostics identified 2 potentially influential effect sizes, from Aridi et al [66] and Kumari et al [93] (Figure S3 in Multimedia Appendix 9). Excluding both simultaneously resulted in a slightly attenuated but statistically significant pooled association (*r*=0.155, 95% CI 0.123-0.186; *P*<.001). Additional sensitivity analyses excluding the exceptionally large sample (N=49,051) [83] or 6 studies classified as low quality (AHRQ<4) [53,82,86,89,100,106] yielded pooled estimates comparable to the primary analysis (Table 1). Overall, the magnitude and statistical significance of the pooled associations were generally consistent.

**Table 1:** Sensitivity analyses of the pooled association between screen time and health outcomes^a^.

Analysis	k	Pooled *r* (95% CI)	τ^2^	*I*^2^ (%)	*P* value
Primary analysis	83	0.175 (0.133-0.217)	0.033	98.35	<.001
Excluding 2 influential effect sizes	81	0.155 (0.123-0.186)	0.019	97.25	<.001
Excluding exceptionally large sample	82	0.177 (0.135-0.219)	0.033	97.82	<.001
Excluding low-quality studies (AHRQ^b^<4)	76	0.183 (0.137-0.228)	0.035	98.54	<.001

^a^Pooled effects were estimated using random-effects models with restricted maximum likelihood estimation and the Hartung-Knapp-Sidik-Jonkman adjustment. AHRQ indicates the study quality assessment score. The exceptionally large sample had N=49,051. The 2 influential effect sizes were from Aridi et al [66] and Kumari et al [93].

^b^AHRQ: Agency for Healthcare Research and Quality.

#### Subgroup Analysis by Health Outcome Type

In subgroup analyses by health outcome type, screen time was significantly associated with both mental health outcomes across 38 independent samples from 37 studies (k=38; *r*=0.176, 95% CI 0.124-0.227; *P*<.001) [17,52,57,68,71,72,74,75,77,79,82, 86,91,94,95,97-99,102,104,108-111,116,117,119-125,129-132] and physical health outcomes (k=45; *r*=0.177, 95% CI 0.108-0.243; *P*<.001) [53-56,58-67,69,70,73,76,78,80,81,83-85, 87-90,92,93,96,100,101,103,105-107,112-115,118,126-128]. Considerable heterogeneity was observed within both the mental health subgroup (*I*^2^=97.64%; τ^2^=0.025; Q_37_=2834.83; *P*<.001) and the physical health subgroup (*I*^2^=98.49%; τ^2^=0.044; Q_44_=1067.46; *P*<.001). However, health outcome type was not a significant moderator of the association between screen time and health outcomes (*F*_1,81_=0.003; *P*=.96), with no heterogeneity explained by this moderator (*R*^2^=0.00%; Figure 3 [17,52-132]).

**Figure 3:**
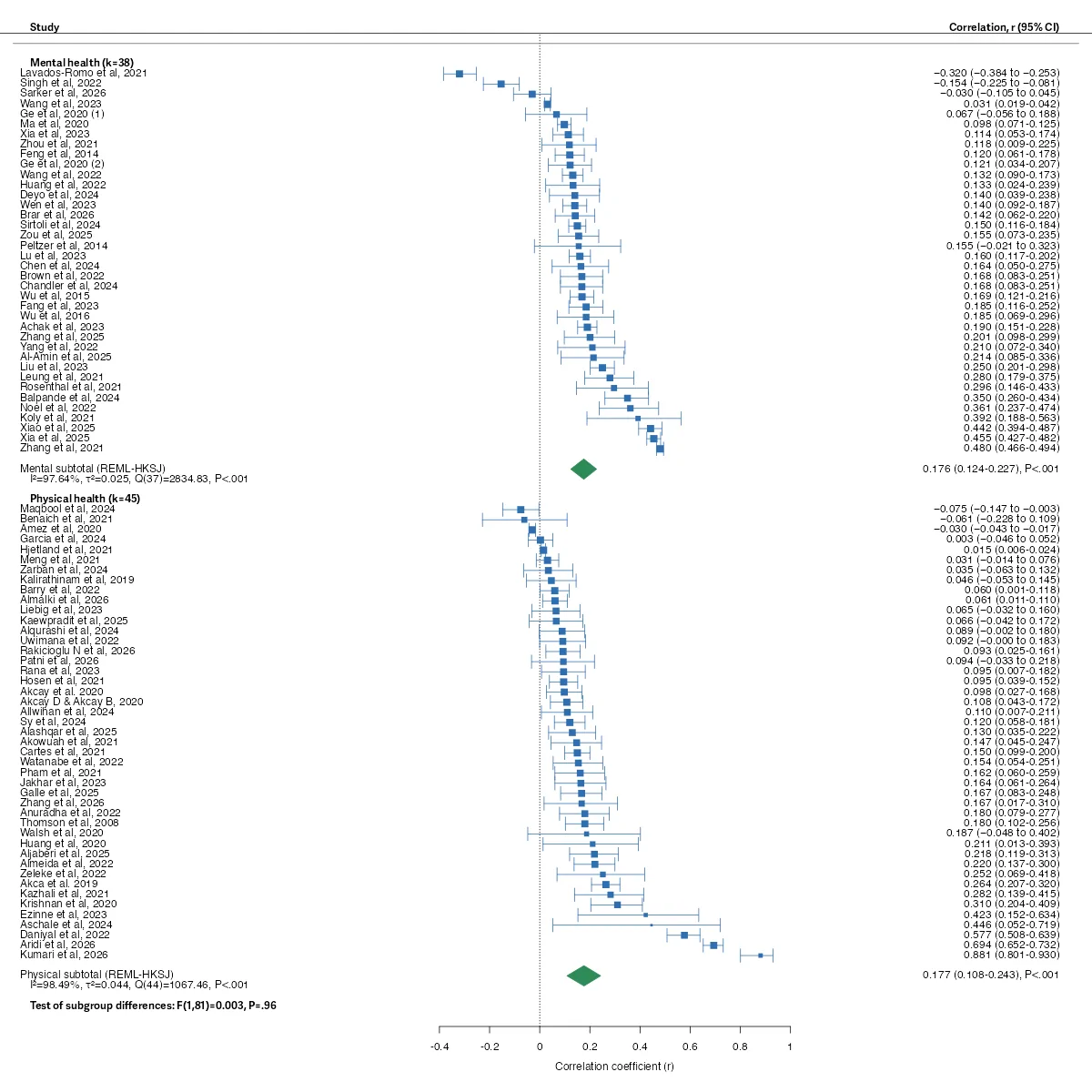
Forest plot of the association between screen time and health outcomes, stratified by mental and physical health domains. HKSJ: Hartung-Knapp-Sidik-Jonkman; REML: restricted maximum likelihood.

#### Subgroup Analysis by Specific Health Outcomes

Among mental health outcomes, the pooled association was statistically significant for depression (k=21; *r*=0.230, 95% CI 0.167-0.291; *P*<.001) [17,57,68,74,86,91,95,104,108,111,116, 117,119-122,124,129-132] and combined mental health outcomes (k=8; *r*=0.170, 95% CI 0.076-0.261; *P*=.004) [17,77,97,98,102,109,123,125], but not for well-being (k=7; *r*=0.040, 95% CI –0.146 to 0.223; *P*=.62) [52,71,72,75,94,99,110] or stress (k=2; *r*=0.103, 95% CI –0.222 to 0.407; *P*=.16) [82]. Considerable heterogeneity was observed for depression (*I*^2^=97.53%; τ^2^=0.019; Q_20_=2461.00; *P*<.001), combined mental health outcomes (*I*^2^=89.10%; τ^2^=0.011; Q_7_=50.69; *P*<.001) and well-being (*I*^2^=97.92%; τ^2^=0.040; Q_6_=202.55; *P*<.001), whereas heterogeneity was not significant for stress (*I*^2^=0.00%; τ^2^=0.000; Q_1_=0.50; *P*=.48). The omnibus moderator test indicated that the magnitude of the association differed significantly across mental health outcomes (*F*_3,34_=3.27; *P*=.03), with mental health outcome type accounting for 15.76% of the heterogeneity. Post hoc pairwise comparisons with Holm adjustment showed that the association was significantly stronger for depression than for well-being (Fisher *z* difference=0.195; Holm-adjusted *P*=.03). No other pairwise comparisons were statistically significant (all Holm-adjusted *P*≥.44). Substantial residual heterogeneity remained after accounting for specific mental health outcome type (*I*^2^=97.04%; τ^2^=0.021; Q_34_=2714.75; *P*<.001; Figure 4 [17,52,57,68,71, 72,74,75,77,79,82,86,91,94,95,97-99,102,104,108-111,116,117, 119-125,129-132]).

**Figure 4:**
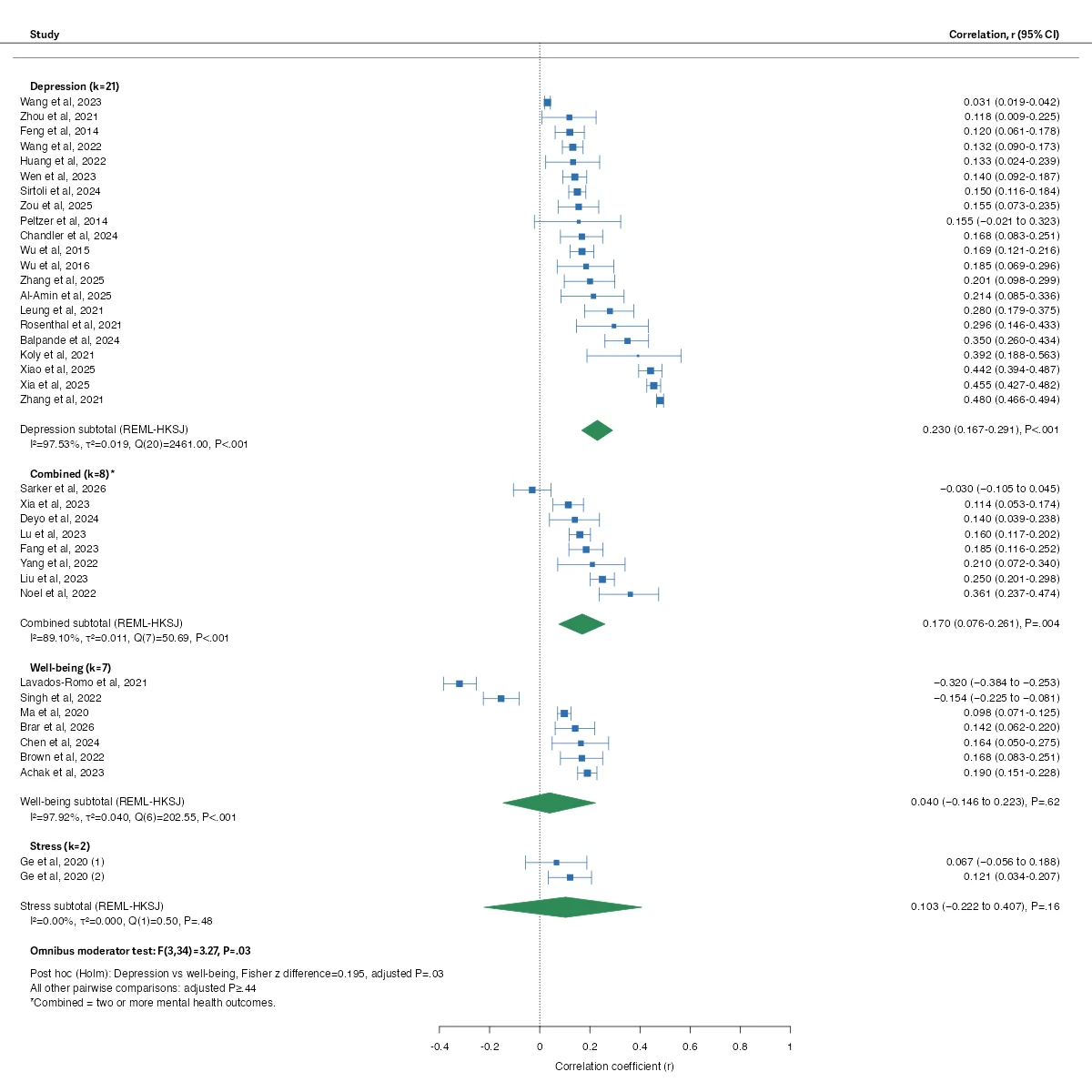
Forest plot of subgroup analyses examining the association between screen time and specific mental health outcomes among university students. Combined mental health outcomes refer to studies assessing 2 or more mental health outcomes. HKSJ: Hartung-Knapp-Sidik-Jonkman; REML: restricted maximum likelihood.

Among physical health outcomes, screen time was significantly associated with sleep quality (k=26; *r*=0.120, 95% CI 0.083-0.158; *P*<.001) [53-56,61,62,64,65,67,69,80,81,83-85, 88,90,92,96,101,105,106,112,115,118,128] and eye health (k=16; *r*=0.277, 95% CI 0.089-0.446; *P*=.007) [58-60,63,66, 73,76,78,87,93,100,103,107,114,126,127], whereas the association with weight-related outcomes was not statistically significant (k=3; *r*=0.071, 95% CI –0.222 to 0.351; *P*=.41) [70,89,113]. Given variation in the measurement of eye health outcomes (4 studies [60,76,87,103] used an established, validated eye health instrument in its original standard form), a post hoc sensitivity analysis restricted to studies using established, validated eye-health instruments in their standard form yielded a similar and statistically significant association (k=12; *r*=0.288, 95% CI 0.038-0.503; *P*=.03; Table S8 in Multimedia Appendix 9). Considerable heterogeneity was observed for sleep quality (*I*^2^=93.50%; τ^2^=0.007; Q_25_=279.16; *P*<.001), eye health (*I*^2^=98.09%; τ^2^=0.123; Q_15_=527.68; *P*<.001), and weight-related outcomes (*I*^2^=77.34%; τ^2^=0.010; Q_2_=8.58; *P*=.01). However, the omnibus moderator test indicated no significant differences in effect sizes across specific physical health outcomes (*F*_2,42_=2.24; *P*=.12), with outcome type accounting for 6.10% of the between-study heterogeneity. Substantial residual heterogeneity remained (*I*^2^=98.37%; τ^2^=0.041; Q_42_=815.42; *P*<.001; Figure 5 [53-56,58-67,69,70,73, 76,78,80,81,83-85,87-90,92,93,96,100,101,103,105-107,112-115,118,126-128]).

**Figure 5:**
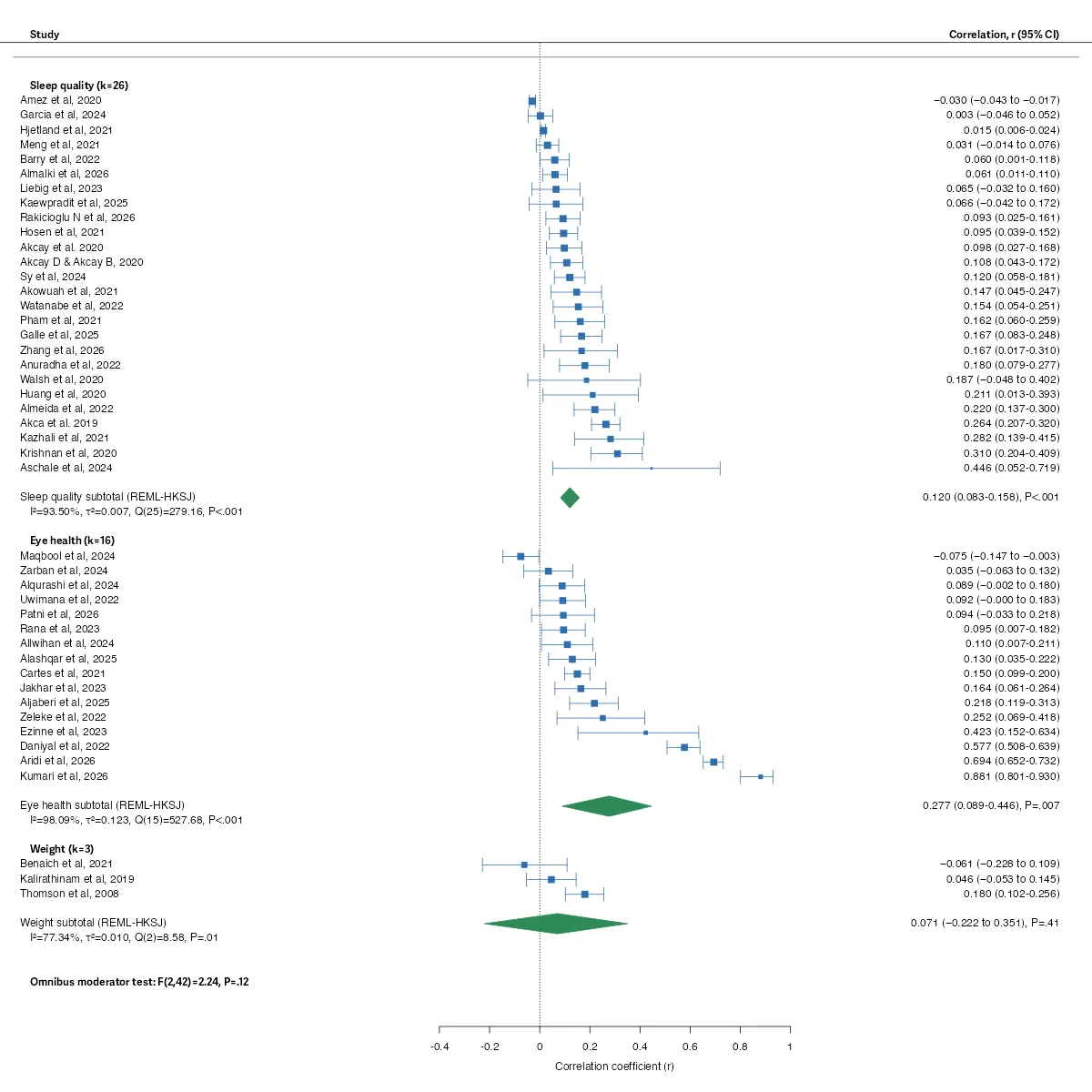
Forest plot of subgroup analyses examining the association between screen time and specific physical health outcomes among university students. HKSJ: Hartung-Knapp-Sidik-Jonkman; REML: restricted maximum likelihood.

#### Meta-Regression Analyses

Exploratory meta-regression analyses were conducted across the overall dataset of 83 independent samples to investigate potential sources of the considerable between-study heterogeneity observed in the primary analysis (*I*^2^=98.35%). None of the examined moderators significantly explained variation in effect sizes, including study design (*F*_1,81_=0.002; *P*=.96; *R*^2^=0.00%), screen time exposure type (*F*_4,78_=0.36; *P*=.84; *R*^2^=0.00%), geographic region (*F*_4,78_=1.53; *P*=.20; *R*^2^=3.49%), proportion of female participants (*β*=–.011 per 10-percentage-point increase, 95% CI –0.037 to 0.016; *P*=.43), study quality (*β*=–.002, 95% CI –0.028 to 0.025; *P*=.90), year of publication (*β*=.008, 95% CI –0.006 to 0.023; *P*=.26), mean participant age (*β*=.007, 95% CI –0.020 to 0.033; *P*=.61). Residual heterogeneity remained statistically significant after adjustment for each moderator (all QE tests; *P*<.001; Table 2).

**Table 2:** Univariable meta-regression analyses of study-level moderators of the association between screen time and health outcomes.

Moderator/comparison			*β* (95% CI)		SE		*P* value		Omnibus *F* test (*df*)		Omnibus *P* value		*R*^2^ (%)
**Study design**									0.002 (1, 81)		.96		0.00
	Cross-sectional	Reference		Reference		Reference							
	Longitudinal	–.004 (–0.189 to 0.181)		0.093		.96							
**Screen time exposure type**									0.36 (4, 78)		.84		0.00
	Total screen time	Reference		Reference		Reference							
	Total social media screen time	.052 (–0.071 to 0.174)		0.062		.41							
	Smartphone screen time	.058 (–0.069 to 0.186)		0.064		.37							
	Recreational screen time	.052 (–0.110 to 0.214)		0.081		.53							
	Computer screen time	.008 (–0.231 to 0.247)		0.120		.95							
**Geographic region**									1.53 (4, 78)		.20		3.49
	Asia	Reference		Reference		Reference							
	North America	–.013 (–0.144 to 0.119)		0.066		.85							
	Others	–.006 (–0.137 to 0.126)		0.066		.93							
	Europe	–.109 (–0.254 to 0.036)		0.073		.14							
	South America	–.205 (–0.401 to –0.009)		0.099		.04							
Proportion of female participants^a^			–.011 (–0.037 to 0.016)		0.013		.43		0.62 (1, 81)		.43		0.00
Study quality			–.002 (–0.028 to 0.025)		0.013		.90		0.02 (1, 81)		.90		0.00
Year of publication^b^			.008 (–0.006 to 0.023)		0.007		.26		1.28 (1, 81)		.26		0.00
Age^c^			.007 (–0.020 to 0.033)		0.007		.61		0.26 (1, 59)		.61		0.00

^a^The coefficient represents the change in Fisher *z* associated with a 10-percentage-point increase in the proportion of female participants.

^b^Year of publication was mean centered at 2022.17 before analysis; centering does not affect the slope estimate or its statistical significance.

^c^Age was mean participant age.

## Discussion

### Principal Findings

This systematic review and meta-analysis synthesizes evidence from 82 studies (83 independent samples) including 161,340 participants to quantify the associations between screen time, physical health, and mental health among university students. This study provides valuable insights into patterns of screen use and their associations with health outcomes among university students, which may help inform future research and the development of strategies addressing screen-related health concerns in this population.

The analysis revealed a statistically significant association of small to typical magnitude between greater screen time and poorer health outcomes among university students. The direction and magnitude of this association are broadly consistent with previous evidence from younger populations. A meta-analysis of children aged 12 years or younger reported small correlations between greater screen time and internalizing (*r*=0.07) and externalizing (*r*=0.11) problems [34]. However, between-study heterogeneity was particularly high in this analysis (*I*^2^=98.35%; Q_82_=4744.45; *P*<.001). Considerable heterogeneity has also been reported in previous meta-analyses of screen time and health outcomes. For example, Eirich et al [34] reported *I*^2^ values of 85.27% and 87.80% for internalizing and externalizing problems, respectively. Although the heterogeneity observed in this review was numerically higher, direct comparisons should be made cautiously because previous reviews differed in their exposure definitions, health outcomes, and populations. The particularly high heterogeneity observed here may therefore reflect, at least partly, the broader scope of this synthesis rather than a characteristic unique to university students. Accordingly, the pooled effect should be interpreted as an average association across heterogeneous study contexts rather than as a uniform effect across the university student population.

The meta-regression analysis was used to assess the effects of the measured covariates: study design, screen time exposure type, geographic area, mean participant age, year of publication, percentage of female individuals, study quality. These broad study-level characteristics were not statistically significantly associated with effect-size variation. However, study-level meta-regression relies on aggregate study characteristics and may have limited ability to identify participant-level sources of heterogeneity because associations observed across studies may not reflect those occurring within studies [133]. Future individual participant data meta-analyses may allow more precise examination of potential moderators at the individual level, such as age, gender, and patterns of screen use [133]. Heterogeneity may also reflect proximal characteristics of screen use that were not consistently captured across studies. Total duration alone may obscure meaningful differences in the timing, purpose, content, and context of screen use [134]. Moreover, heterogeneity may have been compounded by variation in exposure measurement, as all included studies relied on self-reported screen time but used different instruments and scales. Such measures may overestimate or underestimate actual screen time compared with objective measurement such as device-based smartphone tracking, which can provide more direct estimates of screen use [135].

The pooled estimates for mental and physical health outcomes were similar in magnitude, and there was no statistical evidence that health outcome domain moderated the association between screen time and health. This suggests that associations were not clearly confined to either the mental or physical health domain. Nevertheless, similar effect sizes do not necessarily imply common underlying mechanisms. Previous research suggests several possible psychosocial and behavioral pathways for mental health outcomes. Certain forms of screen use, particularly social media use, may facilitate upward social comparison and negative self-evaluation, which have been associated with depressive and anxiety symptoms [136]. These screen-based activities may also be used to cope with psychological distress or may displace face-to-face social interaction, physical activity, and other health-promoting behaviors [108]. For physical health outcomes, the relevant pathways may be more outcome-specific [19]. Evening screen exposure and cognitively stimulating screen activities close to bedtime may contribute to circadian disruption, increased arousal, and delayed sleep onset [137], whereas prolonged visual exposure and sedentary behavior may be more relevant to eye health [138,139] and weight-related outcomes [76], respectively. However, these mechanisms were not directly examined in the included studies and should therefore be regarded as possible explanations rather than established pathways. Substantial heterogeneity also remained within both subgroups, indicating that the broad distinction between mental and physical health does not adequately capture the variability in screen-related health associations. This residual heterogeneity highlights the need to examine more specific outcome, exposure, and measurement characteristics.

A more granular examination of mental health outcomes showed variation across psychological constructs, with the association being significantly stronger for depression than for well-being after adjustment for multiple comparisons. This finding is conceptually consistent with the dual-continua model of mental health, which considers psychological well-being and mental illness to be related but distinct dimensions rather than opposite ends of a single continuum [140,141]. Viewed within this framework, screen time exposure may be more strongly associated with depressive symptoms than with positive psychological functioning. However, these findings do not establish why these associations differ across outcomes. A similar pattern has been observed in previous meta-analytic research on digital media use, which found a positive association with depression but little overall association with aggregated well-being [142]. Moreover, our finding for depression is broadly consistent with a recent meta-analysis of children and adolescents, which reported a positive association between screen time and depressive symptoms [143].

As for specific physical health outcomes, greater screen time was significantly associated with poorer sleep quality and eye health outcomes, whereas the association with weight-related outcomes was not statistically significant. Although the point estimate was numerically larger for eye health outcomes than for sleep quality or weight-related outcomes, the omnibus moderator test did not indicate significant differences across physical health outcome types. One possible explanation is that the relevant pathways differ across outcomes. Screen exposure may be more closely associated with sleep disruption and visual strain [144,145], whereas weight-related outcomes are influenced by a broader range of behavioral and metabolic factors [146]. Importantly, the association with eye health outcomes remained similar in magnitude in a post hoc sensitivity analysis restricted to studies that established validated eye-health instruments in their standard form, suggesting that the pooled estimate was not materially affected by differences in measurement standardization. Given that only 3 effect sizes contributed to the weight-related outcomes subgroup, the nonsignificant finding should not be interpreted as evidence of no association. The GRADE assessment further indicated that the certainty of evidence varied across health outcomes and was primarily limited by substantial inconsistency, with additional concerns regarding methodological quality and imprecision for outcomes. These findings should therefore be interpreted with appropriate caution, particularly for outcomes supported by low-certainty evidence.

### Strengths and Limitations

The review has several notable strengths. First, it provides a comprehensive synthesis of the association between screen time and both mental and physical health outcomes specifically among university students. By focusing on this group, the study addresses an important gap in the existing literature, which has predominantly examined adolescents or general adult populations. Second, the inclusion of 83 independent samples comprising more than 160,000 participants enhanced statistical power. Third, the study considered a broad range of health outcomes and applied a hierarchical subgroup approach, allowing associations to be examined not only across mental and physical health domains but also across more specific outcomes, including depression, stress, well-being, sleep quality, weight-related outcomes, and eye health. Finally, by examining multiple health domains within a single analytic framework, this review offers a comprehensive and comparative evaluation of screen time-related health correlates, thereby contributing more integrated evidence to inform future research and digital health strategies in university settings.

Nevertheless, several limitations should be acknowledged. First, the reported effect sizes represent associations and should not be interpreted as evidence of causality, particularly because most included studies were cross-sectional. Second, screen time was assessed using self-reported measures across the included studies. Such measures are susceptible to recall and reporting error, and considerable variation in screen time definitions, recall periods, devices, and assessment instruments may have contributed to the substantial heterogeneity observed. Third, eye health outcomes were mainly based on self-reported symptoms, with limited objective ophthalmological assessment. The pooled estimate should therefore be interpreted as reflecting ocular symptom burden rather than clinically diagnosed eye disease. Fourth, some outcome subgroups contained few studies, particularly for weight-related outcomes, and it is possible that nonsignificant between-group differences may reflect low statistical power. Fifth, data extraction was conducted by a single reviewer, which may increase the risk of extraction error. Sixth, corresponding authors were not systematically contacted for unreported quantitative data. Therefore, some potentially eligible evidence may have been unavailable because of incomplete reporting. Finally, the GRADE assessment indicated that certainty was reduced primarily because of substantial inconsistency and, for selected outcomes, concerns regarding risk of bias and imprecision.

### Future Research

Given these limitations, future research should aim to include more longitudinal and experimental studies to explore causal relationships between screen time and health outcomes. Second, future studies should use objective measures such as smartphone or device-based tracking to reduce recall and reporting errors and enable more precise characterization of screen exposure. Where relevant, objective clinical assessments should also complement self-reported health outcomes, particularly for eye health. Importantly, future studies should adopt a more differentiated approach by examining screen content (eg, social media and violent video games), context (eg, active or passive viewing), and quality (eg, educational vs entertainment). In addition, they should also distinguish positive mental health from symptoms of mental ill health rather than treating mental health as a single homogeneous construct, given the outcome-specific differences observed in this review.

### Implication

The findings of this review have important implications for both research and practice. From a research perspective, future studies should prioritize longitudinal and prospective designs to clarify temporal relationships and strengthen causal inference. The incorporation of objective screen use metrics (eg, passive sensing technologies) alongside validated self-report instruments may improve measurement precision. Greater standardization in defining and operationalizing screen time, including distinctions between active and passive use, would also enhance comparability across studies [147]. From a practical standpoint, given the widespread and sustained exposure to digital devices among university students, institutions may consider integrating digital literacy, balanced media use guidance, and sleep support interventions into student wellbeing initiatives.

### Conclusions

This systematic review and meta-analysis demonstrated a statistically significant positive association between greater screen time and poorer health outcomes among university students. Associations were evident across both mental and physical health domains, with particularly consistent evidence for depression, sleep quality, and eye health. The pooled association remained robust across multiple sensitivity analyses, but substantial heterogeneity was observed. Future research should use longitudinal and more rigorous and standardized approaches to measuring and analyzing screen exposure and should further examine the content and context of screen use that may influence university students’ health outcomes in an increasingly digital environment.

## Appendix

Multimedia Appendix 1 PRISMA 2020 checklist.

Multimedia Appendix 2 Search strategy.

Multimedia Appendix 3 PRISMA-S (Preferred Reporting Items for Systematic Reviews and Meta-Analyses literature search extension).

Multimedia Appendix 4 Inclusion and exclusion criteria.

Multimedia Appendix 5 Study and sample characteristics. Measurement characteristics of studies included in the eye health outcome category.

Multimedia Appendix 6 Study quality evaluation coding criteria and study quality scoring for each study included in the meta-analysis.

Multimedia Appendix 7 GRADE certainty of evidence for the association between screen time and health outcomes.

Multimedia Appendix 8 Funnel plot assessing small sample study effect in the overall association between screen time and health outcomes among university students.

Multimedia Appendix 9 Leave-one-out sensitivity analysis of the association between screen time and health outcomes; influence diagnostics for the overall random-effects meta-analysis of the association between screen time and health outcomes among university students; and sensitivity analyses of the associations between screen time and eye health outcomes.

## Abbreviations

AHRQ: Agency for Healthcare Research and Quality
CMA: Comprehensive Meta-Analysis
GRADE: Grading of Recommendations Assessment, Development, and Evaluation
HKSJ: Hartung-Knapp-Sidik-Jonkman
PRISMA: Preferred Reporting Items for Systematic Reviews and Meta-Analysis
PRISMA-S: Preferred Reporting Items for Systematic Reviews and Meta-Analyses Search
PROSPERO: International Prospective Register of Systematic Reviews
REML: restricted maximum likelihood

## References

[1] Stiglic N, Viner RM (2019). Effects of screentime on the health and well-being of children and adolescents: a systematic review of reviews. BMJ Open.

[2] LeBlanc AG, Gunnell KE, Prince SA, Saunders TJ, Barnes JD, Chaput J (2017). The ubiquity of the screen: an overview of the risks and benefits of screen time in our modern world. Transl J Am Coll Sports Med.

[3] Hale L, Guan S (2015). Screen time and sleep among school-aged children and adolescents: a systematic literature review. Sleep Med Rev.

[4] Tremblay MS, LeBlanc AG, Kho ME, Saunders TJ, Larouche R, Colley RC, Goldfield G, Connor Gorber S (2011). Systematic review of sedentary behaviour and health indicators in school-aged children and youth. Int J Behav Nutr Phys Act.

[5] Twenge JM, Campbell WK (2018). Associations between screen time and lower psychological well-being among children and adolescents: evidence from a population-based study. Prev Med Rep.

[6] Primack BA, Carroll MV, McNamara M, Klem ML, King B, Rich M, Chan CW, Nayak S (2012). Role of video games in improving health-related outcomes: a systematic review. Am J Prev Med.

[7] Mertens G, Gerritsen L, Duijndam S, Salemink E, Engelhard IM (2020). Fear of the coronavirus (COVID-19): predictors in an online study conducted in March 2020. J Anxiety Disord.

[8] Oyeyemi AL, Muhammed S, Oyeyemi AY, Adegoke BOA (2017). Patterns of objectively assessed physical activity and sedentary time: are Nigerian health professional students complying with public health guidelines?. PLoS One.

[9] Rouse PC, Biddle SJH (2010). An ecological momentary assessment of the physical activity and sedentary behaviour patterns of university students. Health Educ J.

[10] Mussi FC, Pitanga FJG, Pires CGDS (2017). Cumulative sitting time as discriminator of overweight, obesity, abdominal obesity and lipid disorders in nursing university. Rev Bras Cineantropom Desempenho Hum.

[11] Clark BK, Pavey TG, Lim RF, Gomersall SR, Brown WJ (2016). Past-day recall of sedentary time: validity of a self-reported measure of sedentary time in a university population. J Sci Med Sport.

[12] Conroy DE, Maher JP, Elavsky S, Hyde AL, Doerksen SE (2013). Sedentary behavior as a daily process regulated by habits and intentions. Health Psychol.

[13] Farinola M, Bazan N (2011). Sedentary behavior and physical activity in university students: a pilot study. Revista argentina de cardiología.

[14] Moulin MS, Irwin JD (2017). An assessment of sedentary time among undergraduate students at a Canadian university. Int J Exerc Sci.

[15] Beiter R, Nash R, McCrady M, Rhoades D, Linscomb M, Clarahan M, Sammut S (2015). The prevalence and correlates of depression, anxiety, and stress in a sample of college students. J Affect Disord.

[16] Chang J, Ji Y, Li Y, Pan H, Su P (2021). Prevalence of anxiety symptom and depressive symptom among college students during COVID-19 pandemic: a meta-analysis. J Affect Disord.

[17] Feng Q, Zhang QL, Du Y, Ye YL, He QQ (2014). Associations of physical activity, screen time with depression, anxiety and sleep quality among Chinese college freshmen. PLoS One.

[18] Jing Z, Yang W, Lei Z, Junmei W, Hui L, Tianmin Z (2025). Correlations between social media addiction and anxiety, depression, FoMO, loneliness and self-esteem among students: a systematic review and meta-analysis. PLoS One.

[19] Zeng Z, Shi Z, Hou S, Yan J, Yang R, Li X (2026). Comprehensive effects and dose-response relationship of screen exposure on university students' physical and mental health and academic performance in the digital age: a systematic review and meta-analysis. BMC Public Health.

[20] Shamseer L, Moher D, Clarke M, Ghersi D, Liberati A, Petticrew M, Shekelle P, Stewart LA, PRISMA-P Group (2015). Preferred Reporting Items for Systematic Review and Meta-Analysis Protocols (PRISMA-P) 2015: elaboration and explanation. BMJ.

[21] Rethlefsen ML, Kirtley S, Waffenschmidt S, Ayala AP, Moher D, Page MJ, Koffel JB, PRISMA-S Group (2021). PRISMA-S: an extension to the PRISMA statement for reporting literature searches in systematic reviews. Syst Rev.

[22] Poitras VJ, Gray CE, Borghese MM, Carson V, Chaput J, Janssen I, Katzmarzyk PT, Pate RR, Connor Gorber S, Kho ME, Sampson M, Tremblay MS (2016). Systematic review of the relationships between objectively measured physical activity and health indicators in school-aged children and youth. Appl Physiol Nutr Metab.

[23] Ouzzani M, Hammady H, Fedorowicz Z, Elmagarmid A (2016). Rayyan-a web and mobile app for systematic reviews. Syst Rev.

[24] Forbes CD (1997). Book review: the pocket guide to critical appraisal. Scott Med J.

[25] Qi J, Liu E, Guo YF, Hu JM, Liu YT, Chen G, Yue HQ (2021). Association between periodontal disease and osteoporosis in postmenopausal women: a protocol for systematic review and meta-analysis. BMJ Open.

[26] Balshem H, Helfand M, Schünemann HJ, Oxman AD, Kunz R, Brozek J, Vist GE, Falck-Ytter Y, Meerpohl J, Norris S, Guyatt GH (2011). GRADE guidelines: 3. Rating the quality of evidence. J Clin Epidemiol.

[27] Schünemann HJ, Brennan S, Akl EA, Hultcrantz M, Alonso-Coello P, Xia J, Davoli M, Rojas MX, Meerpohl JJ, Flottorp S, Guyatt G, Mustafa RA, Langendam M, Dahm P (2023). The development methods of official GRADE articles and requirements for claiming the use of GRADE - a statement by the GRADE guidance group. J Clin Epidemiol.

[28] No authors listed (2025). Chapter C. 7: GRADE criteria determining certainty of evidence. ACIP GRADE Handbook for Developing Evidence-based Recommendations.

[29] Guyatt GH, Oxman AD, Kunz R, Woodcock J, Brozek J, Helfand M, Alonso-Coello P, Glasziou P, Jaeschke R, Akl EA, Norris S, Vist G, Dahm P, Shukla VK, Higgins J, Falck-Ytter Y, Schünemann HJ, GRADE Working Group (2011). GRADE guidelines: 7. Rating the quality of evidence--inconsistency. J Clin Epidemiol.

[30] Guyatt GH, Oxman AD, Kunz R, Woodcock J, Brozek J, Helfand M, Alonso-Coello P, Falck-Ytter Y, Jaeschke R, Vist G, Akl EA, Post PN, Norris S, Meerpohl J, Shukla VK, Nasser M, Schünemann HJ, GRADE Working Group (2011). GRADE guidelines: 8. Rating the quality of evidence--indirectness. J Clin Epidemiol.

[31] Guyatt GH, Oxman AD, Kunz R, Brozek J, Alonso-Coello P, Rind D, Devereaux PJ, Montori VM, Freyschuss B, Vist G, Jaeschke R, Williams JW, Murad MH, Sinclair D, Falck-Ytter Y, Meerpohl J, Whittington C, Thorlund K, Andrews J, Schünemann HJ (2011). GRADE guidelines 6. Rating the quality of evidence--imprecision. J Clin Epidemiol.

[32] Brumariu LE, Madigan S, Giuseppone KR, Movahed Abtahi M, Kerns KA (2018). The Security Scale as a measure of attachment: meta-analytic evidence of validity. Attach Hum Dev.

[33] Caldwell DM, Davies SR, Thorn JC, Palmer JC, Caro P, Hetrick SE, Gunnell D, Anwer S, López-López JA, French C, Kidger J, Dawson S, Churchill R, Thomas J, Campbell R, Welton NJ (2021). School-based interventions to prevent anxiety, depression and conduct disorder in children and young people: a systematic review and network meta-analysis. Public Health Res.

[34] Eirich R, McArthur BA, Anhorn C, McGuinness C, Christakis DA, Madigan S (2022). Association of screen time with internalizing and externalizing behavior problems in children 12 years or younger: a systematic review and meta-analysis. JAMA Psychiatry.

[35] Witzel TC, Eshun-Wilson I, Jamil MS, Tilouche N, Figueroa C, Johnson CC, Reid D, Baggaley R, Siegfried N, Burns FM, Rodger AJ, Weatherburn P (2020). Comparing the effects of HIV self-testing to standard HIV testing for key populations: a systematic review and meta-analysis. BMC Med.

[36] Peterson RA, Brown SP (2005). On the use of beta coefficients in meta-analysis. J Appl Psychol.

[37] Borenstein M, Rothstein H (1999). Comprehensive Meta-Analysis: Biostat.

[38] Welz T, Doebler P, Pauly M (2022). Fisher transformation based confidence intervals of correlations in fixed- and random-effects meta-analysis. Br J Math Stat Psychol.

[39] Viechtbauer W (2010). Conducting meta-analyses in R with the metafor package. J Stat Soft.

[40] IntHout J, Ioannidis JPA, Borm GF (2014). The Hartung-Knapp-Sidik-Jonkman method for random effects meta-analysis is straightforward and considerably outperforms the standard DerSimonian-Laird method. BMC Med Res Methodol.

[41] DerSimonian R, Laird N (1986). Meta-analysis in clinical trials. Control Clin Trials.

[42] Gignac GE, Szodorai ET (2016). Effect size guidelines for individual differences researchers. Pers Individ Dif.

[43] Funder DC, Ozer DJ (2019). Evaluating effect size in psychological research: sense and nonsense. Adv Methods Pract Psychol Sci.

[44] IntHout J, Ioannidis JPA, Rovers MM, Goeman JJ (2016). Plea for routinely presenting prediction intervals in meta-analysis. BMJ Open.

[45] Egger M, Davey Smith G, Schneider M, Minder C (1997). Bias in meta-analysis detected by a simple, graphical test. BMJ.

[46] Lau J, Ioannidis JPA, Terrin N, Schmid CH, Olkin I (2006). The case of the misleading funnel plot. BMJ.

[47] Duval S, Tweedie R (2000). Trim and fill: a simple funnel-plot-based method of testing and adjusting for publication bias in meta-analysis. Biometrics.

[48] Borenstein M, Hedges LV, Higgins JPT, Rothstein HR (2009). Introduction to Meta-Analysis.

[49] Viechtbauer W, Cheung MWL (2010). Outlier and influence diagnostics for meta-analysis. Res Synth Methods.

[50] Higgins J, Thomas J, Chandler J, Cumpston M, Li T, Page M (2019). Cochrane Handbook for Systematic Reviews of Interventions.

[51] Tipton E (2015). Small sample adjustments for robust variance estimation with meta-regression. Psychol Methods.

[52] Achak D, El-Ammari A, Azizi A, Youlyouz-Marfak I, Saad E, Nejjari C, Hilali A, Marfak A (2023). Lifestyle habits determinants of health-related quality of life in Moroccan college students. Int J Environ Res Public Health.

[53] Akca N, Senturk S, Alsac S (2019). The effect of cell phone use in adolescents on sleep quality: central Anatolia case. Int J Caring Sci.

[54] Akçay BD, Akçay D, Yetkin S (2021). The effects of mobile electronic devices use on the sleep states of university students. Alpha Psychiatry.

[55] Akçay D, Akçay BD (2020). The effect of computer game playing habits of university students on their sleep states. Perspect Psychiatr Care.

[56] Akowuah PK, Nti AN, Ankamah-Lomotey S, Frimpong AA, Fummey J, Boadi P, Osei-Poku K, Adjei-Anang J (2021). Digital device use, computer vision syndrome, and sleep quality among an African undergraduate population. Advances in Public Health.

[57] Al-Amin M, Rinky F, Nizamul Hoque Bhuiyan M, Yeasmin R, Akter T, Hoque N, Reza S (2025). Factors influencing mental health outcomes among university students: a cross-sectional study in Bangladesh. BMJ Open.

[58] Alashqar M, Taqatqa S, Ayaseha A, Shanab ARA, Shawahna R (2025). Electronic device exposure and dry eye symptoms among medical and nonmedical Palestinian university students: a multicenter cross-sectional study of associated factors. BMC Ophthalmol.

[59] Aljaberi HA, Rahmani S, Elzahra ZA, Mohammed SH (2025). Prevalence of dry eye symptoms and associated risk factors among students at Al-Mustaqbal University, Iraq. PLoS One.

[60] Allwihan R, Alhalwani AY, Khojah M, Abduljawad JM, Albedaiwi TB, Bazhair RM, Alkhayyat AA, Alsolami MN, Anbari LK (2024). The impact of electronic device use on dry eye disease symptoms based on age and gender: a cross-sectional study in health science university students. TOOPHTJ.

[61] Almalki MA, Ahmed MA, Alzahrani AM, Mehmood A, Felix HC, Almalki R, Alhashemy B, Musawi H (2026). An integrative analysis of behavioral, psychological, and smartphone use factors associated with sleep quality: a cross-sectional study of Saudi undergraduate students. Inquiry.

[62] Almeida F, Marques DR, Gomes AA (2023). A preliminary study on the association between social media at night and sleep quality: the relevance of FOMO, cognitive pre-sleep arousal, and maladaptive cognitive emotion regulation. Scand J Psychol.

[63] Alqurashi A, Almaghrabi H, Alahmadi M, Alotaibi A, Alotaibi B, Jastaniah A, Bukhari A, Binhussein M, Othman B, Khojah A (2024). The severity of dry eye symptoms and risk factors among university students in Saudi Arabia: a cross-sectional study. Sci Rep.

[64] Amez S, Vujić S, Soffers P, Baert S (2020). Yawning while scrolling? Examining gender differences in the association between smartphone use and sleep quality. J Sleep Res.

[65] Anuradha R, Hemachandran S, Patil AB (2022). Sleep quality and daytime sleepiness among medical undergraduate students in Tamil Nadu: a cross-sectional study. JCDR.

[66] Aridi M, Alwan N, Ghach W (2026). Behavioral and cultural determinants of symptomatic dry eye disease among university students in the UAE. PLoS One.

[67] Aschale Wale M, Reta Y, Addis H, Tarekegn R, Tafese M, Tsega Chekol A (2024). Predictors of insomnia among undergraduate students at Hawassa University Sidama, Ethiopia, 2023: a facility-based cross-sectional study. Front Psychiatry.

[68] Balpande LS, Pusdekar YV, Bhagat SS, Patil PD, Saoji AV (2024). Influence of social media on psychological health of youth during COVID-19 pandemic. J Family Med Prim Care.

[69] Barry CT, Moran-Miller K, Levy HF, Gray T (2024). Social media engagement, perceptions of social media costs and benefits, and well-being in college student-athletes. J Am Coll Health.

[70] Benaich S, Mehdad S, Andaloussi Z, Boutayeb S, Alamy M, Aguenaou H, Taghzouti K (2021). Weight status, dietary habits, physical activity, screen time and sleep duration among university students. Nutr Health.

[71] Brar S, King N, Park A, Kyone K, Dephoure E, Rivera D, Lyon A, Duffy A (2026). Recreational screen time at university entry and mental health and well-being over first year: U-Flourish student well-being research: Temps d'écran à des fins de divertissement au moment de l'entrée à l'université, santé mentale et bien-être au cours de la première année : programme de recherche U-Flourish sur le bien-être des étudiants. Can J Psychiatry.

[72] Brown DM, Faulkner GE, Kwan MY (2022). Healthier movement behavior profiles are associated with higher psychological wellbeing among emerging adults attending post-secondary education. J Affect Disord.

[73] Cartes C, Segovia C, Salinas-Toro D, Goya C, Alonso MJ, Lopez-Solis R, Zapata C, Cabezas M, Yañez P, Flores-Rodriguez P, Lopez D, Matus G, Traipe L (2022). Dry eye and visual display terminal-related symptoms among university students during the coronavirus disease pandemic. Ophthalmic Epidemiol.

[74] Chandler MC, Ellison OK, McGowan AL, Fenn KM, Pontifex MB (2024). Physical activity and sleep moderate the relationship between stress and screen time in college-aged adults. J Am Coll Health.

[75] Chen M, Padmapriya N, Chua XH, Escobosa LLT, Tay AE, Tai BC, Petrunoff N, Müller-Riemenschneider Falk (2024). Screen viewing patterns and their association with mental wellbeing and psychological distress: a cross-sectional study amongst university students. BMC Public Health.

[76] Daniyal M, Javaid SF, Hassan A, Khan MAB (2022). The relationship between cellphone usage on the physical and mental wellbeing of university students: a cross-sectional study. Int J Environ Res Public Health.

[77] Deyo A, Wallace J, Kidwell KM (2024). Screen time and mental health in college students: time in nature as a protective factor. J Am Coll Health.

[78] Ezinne N, Alemu HW, Cheklie T, Ekemiri K, Mohammed R, James S (2023). High prevalence of symptomatic dry eye disease among university students during the COVID-19 pandemic in University of West Indies, Trinidad and Tobago. Clin Optom (Auckl).

[79] Fang M, Ma T, Li H, Han T, Wang J, Li Z, Zhou J (2023). Association of the awareness of the epidemic, mental health status with mobile phone screen use time in Chinese college students during COVID-19 isolation and control. PLoS Glob Public Health.

[80] Gallè F, Grassi F, Valeriani F, Albertini R, Angelillo S, Caggiano G, Bargellini A, Bianco A, Bianco L, Dallolio L, Della Polla G, Di Giuseppe G, Gioffrè ME, Laganà P, Licata F, Liguori F, Lo Moro G, Marchesi I, Martella M, Masini A, Montagna MT, Napoli C, Oliva S, Orsi GB, Paduano S, Pasquarella C, Pelullo CP, Sacchetti R, Siliquini R, Triggiano F, Veronesi L, Spica VR, Vitali M, Protano C (2025). Sleep quality among Italian university students: the UnSleep multicenter study. Ann Ig.

[81] Garcia MC, Paravidino VB, Lopes CDS, Mediano MFF, Gonçalves TR, de Oliveira AJ, Sichieri R (2024). Sleep duration and quality during the COVID-19 pandemic and the association with physical activity and screen time among Brazilian college students. Am J Hum Biol.

[82] Ge Y, Xin S, Luan D, Zou Z, Bai X, Liu M, Gao Q (2020). Independent and combined associations between screen time and physical activity and perceived stress among college students. Addict Behav.

[83] Hjetland GJ, Skogen JC, Hysing M, Sivertsen B (2021). The association between self-reported screen time, social media addiction, and sleep among Norwegian university students. Front Public Health.

[84] Hosen MJ, Eva SA, Rahman MM, Ibrahim M, Lira UF, Hossain AB, Shill MC, Uddin MJ (2021). Health impacts of excessive use of Facebook among university students in Bangladesh. Heliyon.

[85] Huang Q, Li Y, Huang S, Qi J, Shao T, Chen X, Liao Z, Lin S, Zhang X, Cai Y, Chen H (2020). Smartphone use and sleep quality in Chinese college students: a preliminary study. Front Psychiatry.

[86] Huang T, Zheng K, Li S, Yang Y, Kong L, Zhao Y (2022). Screen-based sedentary behaviors but not total sedentary time are associated with anxiety among college students. Front Public Health.

[87] Jakhar F, Rodrigues G, Mendonca T, Nayak R, Kamath G, Kamath S, Kamath A (2023). Dry eye symptoms and digital eyestrain - emerging epidemics among university students due to online curriculum amid the COVID-19 pandemic. A cross-sectional study. Indian J Ophthalmol.

[88] Kaewpradit K, Ngamchaliew P, Buathong N (2025). Digital screen time usage, prevalence of excessive digital screen time, and its association with mental health, sleep quality, and academic performance among southern university students. Front Psychiatry.

[89] Kalirathinam D, Hui TX, Jacob S, Sadagobane SK, Chellappan ME (2019). Association between screen time and body mass index among university students. Sci Med.

[90] Al Kazhali M, Shahwan M, Hassan N, Jairoun A (2023). Social media use is linked to poor sleep quality: the opportunities and challenges to support evidence-informed policymaking in the UAE. J Public Health (Oxf).

[91] Koly KN, Sultana S, Iqbal A, Dunn JA, Ryan G, Chowdhury AB (2021). Prevalence of depression and its correlates among public university students in Bangladesh. J Affect Disord.

[92] Krishnan B, Sanjeev RK, Latti RG (2020). Quality of sleep among bedtime smartphone users. Int J Prev Med.

[93] Kumari S, Maran YM, Krishnan KA, Raja M, Paraman V (2026). Digital eye strain among Indian university students in the post-COVID era: a cross-sectional study. Front Public Health.

[94] Lavados-Romo P, Andrade-Mayorga O, Morales G, Muñoz S, Balboa-Castillo T (2023). Association of screen time and physical activity with health-related quality of life in college students. J Am Coll Health.

[95] Leung ANM, Law W, Liang YY, Au ACL, Li C, Ng HKS (2021). What explains the association between usage of social networking sites (SNS) and depression symptoms? The mediating roles of self-esteem and fear of missing out. Int J Environ Res Public Health.

[96] Liebig L, Bergmann A, Voigt K, Balogh E, Birkas B, Faubl N, Kraft T, Schöniger K, Riemenschneider H (2023). Screen time and sleep among medical students in Germany. Sci Rep.

[97] Liu Y, Sun Y, Zheng K, Zheng J, Kong L, Gu J, Huang T (2023). Association of screen time with anxiety and depressive symptoms in college students during COVID-19 outbreak in Shanghai: mediation role of sleep quality. Cyberpsychol Behav Soc Netw.

[98] Lu H, Liang G, Li DL, Liu MX, Yin ZJ, Li YZ, Zhang T, Pan CW (2023). Sleep quality as a mediator of the relationship between screen time and negative emotions among Chinese college freshmen. Psychol Health Med.

[99] Ma C, Zhou L, Xu W, Ma S, Wang Y (2020). Associations of physical activity and screen time with suboptimal health status and sleep quality among Chinese college freshmen: a cross-sectional study. PLoS One.

[100] Maqbool S (2024). Computer vision syndrome among college students: an emerging health issue of current era. Pak J Public Health.

[101] Meng J, Wang F, Chen R, Hua H, Yang Q, Yang D, Wang N, Li X, Ma F, Huang L, Zou Z, Li M, Wang T, Luo Y, Li Y, Liu Y (2021). Association between the pattern of mobile phone use and sleep quality in Northeast China college students. Sleep Breath.

[102] Noel JK, Jacob S, Wensley IA, Rosenthal SR (2022). Subjective smartphone screen time and co-morbid mental illness. J Technol Behav Sci.

[103] Patni MA, Fatima W, Alshamsi MA, Jama HA, Shafeeq M, Rajiv R, Alsoued MS, George BT, Salama RAA, Elamin AAE, Pandurangan T, Bahutair SN, Ahmad H (2026). Digital eye health and behavioral determinants of screen use among university students in the UAE. Front Digit Health.

[104] Peltzer K, Pengpid S, Apidechkul T (2014). Heavy internet use and its associations with health risk and health-promoting behaviours among Thai university students. Int J Adolesc Med Health.

[105] Pham HT, Chuang H, Kuo C, Yeh T, Liao W (2021). Electronic device use before bedtime and sleep quality among university students. Healthcare (Basel).

[106] Rakıcıoğlu N, Yeniçağ Emre R, Dedebayraktar D (2026). Screen time, internet use, and lifestyle habits among health and engineering students: a cross-sectional study. Hacettepe Univ Fac Health Sci J.

[107] Rana M, Gupta PC, Grover S, Furr A, Bhargava N (2023). Prevalence and association of digital eye strain with the quality of sleep and feeling of loneliness among female college students in Northern India. Indian J Public Health.

[108] Rosenthal SR, Zhou J, Booth ST (2021). Association between mobile phone screen time and depressive symptoms among college students: a threshold effect. Hum Behav Emerg Technol.

[109] Sarker MJA, Maranto R, Haque A, Sagar MSU (2026). Social media impacts on Bangladeshi college students. Discov Psychol.

[110] Singh S, Balhara YPS, Kattula D, Ganesh R, Bhargava R, Abhijita B, Gupta A, Gupta A (2022). Impact of COVID-19 pandemic on screen time: findings from a cross-sectional observational study among college students from India. J Glob Issues.

[111] Sirtoli R, Fernández-Rodríguez R, Balboa-Castillo T, Rodrigues R, Garrido-Miguel M, Eumann Mesas A, Morales G, Molino Guidoni C (2024). Time spent on social media and depressive symptoms in university students: the mediating role of psychoactive substances. Am J Addict.

[112] Sy JRT, Dietch JR, Brombach RK, Trevorrow T, Zhou ES (2025). Screen time and insomnia among college students: the moderating effect of loneliness. J Am Coll Health.

[113] Thomson M, Spence JC, Raine K, Laing L (2008). The association of television viewing with snacking behavior and body weight of young adults. Am J Health Promot.

[114] Uwimana A, Ma C, Ma X (2022). Concurrent rising of dry eye and eye strain symptoms among university students during the COVID-19 pandemic era: a cross-sectional study. Risk Manag Healthc Policy.

[115] Walsh NA, Rodriguez N, Repa LM, King E, Garland SN (2020). Associations between device use before bed, mood disturbance, and insomnia symptoms in young adults. Sleep Health.

[116] Wang J, Hao QH, Peng W, Tu Y, Zhang L, Zhu TM (2023). Relationship between smartphone addiction and eating disorders and lifestyle among Chinese college students. Front Public Health.

[117] Wang J, Li W, Ding L, Chen S (2022). The relationship between duration of smartphone uses and anxiety in university students during the COVID-19 outbreak. Int J Environ Res Public Health.

[118] Watanabe K, Adachi H, Yamamoto R, Fujino R, Ishimaru D, Kanayama D, Sakagami Y, Akamine S, Marutani N, Mamiya Y, Mashita M, Nakano N, Kudo T, Ikeda M (2022). Increased digital media use is associated with sleep problems among university students: a study during the COVID-19 pandemic in Japan. Front Psychiatry.

[119] Wen X, Cai Y, Li K, Wang Z, Zhang W, Qin M (2023). A cross-sectional association between screen-based sedentary behavior and anxiety in academic college students: mediating role of negative emotions and moderating role of emotion regulation. Psychol Res Behav Manag.

[120] Wu X, Tao S, Zhang S, Zhang Y, Chen K, Yang Y, Hao J, Tao F (2016). Impact of screen time on mental health problems progression in youth: a 1-year follow-up study. BMJ Open.

[121] Wu X, Tao S, Zhang Y, Zhang S, Tao F (2015). Low physical activity and high screen time can increase the risks of mental health problems and poor sleep quality among Chinese college students. PLoS One.

[122] Xia LL, Li JD, Duan F, Zhang J, Mu LL, Wang LJ, Jiao CY, Song X, Wang Z, Chen JX, Wang JJ, Wang Y, Zhang XC, Jiao D (2023). Effects of online game and short video behavior on academic delay of gratification - mediating effects of anxiety, depression and retrospective memory. Psychol Res Behav Manag.

[123] Xia Z, Tan TC, Liu Y, Zheng J, Li Z, Gu Q, Huang T (2025). Sedentary behaviors negatively related to depressive symptoms in college students: the roles of type and duration of sedentary behaviors. Physiol Behav.

[124] Xiao L, Xiao S, Wang J, Zhang Y, Zhang C, Yan Y (2025). Electronic device use and depressive symptoms in college students mediated by sleep onset time. Sci Rep.

[125] Yang BW, Zou P, Chen Q, Sun L, Ling X, Yang H, Zhou NY, Wang LH, Huang LP, Liu JY, Yang HF, Cao J, Ao L (2022). Lifestyle-related risk factors correlated with mental health problems: a longitudinal observational study among 686 male college students in Chongqing, China. Front Public Health.

[126] Zarban NA, Alammari OB, Abu Sabah S, Alshamrani NSM, Alqathanin MA, AlRabeeah NA, Basalib SG (2024). Prevalence and risk factors of dry eye disease in association with the increased use of electronic devices among university students in western Saudi Arabia. Cureus.

[127] Zeleke TC, Adimassu NF, Alemayehu AM, Dawud TW, Mersha GA (2022). Symptomatic dry eye disease and associated factors among postgraduate students in Ethiopia. PLoS One.

[128] Zhang J, Wang Z, Xu W, Jiang R, Zheng W, Kang W, Feng M, Zhang Y, Lu P (2026). Bedtime electronic device use and sleep quality in Chinese young adults: a longitudinal study. BMC Public Health.

[129] Zhang L, Zhao S, Zhao S, Ke Y, Yang W, Lei M (2025). Association between the time of exposure to electronic screen and anxiety and depression after controlling demographics, exercises, and lifestyles among university students. Neuropsychiatr Dis Treat.

[130] Zhang Y, Wu X, Tao S, Li S, Ma L, Yu Y, Sun G, Li T, Tao F (2021). Associations between screen time, physical activity, and depressive symptoms during the 2019 coronavirus disease (COVID-19) outbreak among Chinese college students. Environ Health Prev Med.

[131] Zhou H, Dai X, Lou L, Zhou C, Zhang W (2021). Association of sedentary behavior and physical activity with depression in sport university students. Int J Environ Res Public Health.

[132] Zou Q, Qin J, Zhang L, Wang W, Yuan X, Wang T, Peng Y, Chen Y, Dou L, Yang X, Abudurexiti X, Wu M (2025). Time spent on social media applications in relation to depressive symptoms during emerging adulthood and the mediating role of sleep quality: cross-sectional observational study. J Med Internet Res.

[133] Riley RD, Lambert PC, Abo-Zaid G (2010). Meta-analysis of individual participant data: rationale, conduct, and reporting. BMJ.

[134] Shannon H, Bush K, Villeneuve PJ, Hellemans KG, Guimond S (2022). Problematic social media use in adolescents and young adults: systematic review and meta-analysis. JMIR Ment Health.

[135] Hodes LN, Thomas KG (2021). Smartphone screen time: inaccuracy of self-reports and influence of psychological and contextual factors. Comput Hum Behav.

[136] Bergagna E, Tartaglia S (2018). Self-esteem, social comparison, and Facebook use. Eur J Psychol.

[137] Esubalew D, Delie AM, Limenh LW, Worku NK, Fenta ET, Hailu M, Abie A, Mehari MG, Dagnaw TE, Melese M (2024). Poor sleep quality among bedtime smartphone user medical students in Ethiopia, 2024. Sleep Med X.

[138] Fernandez-Villacorta D, Soriano-Moreno AN, Galvez-Olortegui T, Agui-Santivañez N, Soriano-Moreno DR, Benites-Zapata VA (2021). Computer visual syndrome in graduate students of a private university in Lima, Perú. Arch Soc Esp Oftalmol (Engl Ed).

[139] Abudawood GA, Ashi HM, Almarzouki NK (2020). Computer vision syndrome among undergraduate medical students in King Abdulaziz University, Jeddah, Saudi Arabia. J Ophthalmol.

[140] Keyes CLM (2005). Mental illness and/or mental health? Investigating axioms of the complete state model of health. J Consult Clin Psychol.

[141] Iasiello M, van Agteren J, Muir Cochrane E (2020). Mental health and/or mental illness: a scoping review of the evidence and implications of the dual-continua model of mental health. Evid Base.

[142] Hancock J, Liu SX, Luo M, Mieczkowski H (2022). Psychological well-being and social media use: a meta-analysis of associations between social media use and depression, anxiety, loneliness, eudaimonic, hedonic and social well-being. SSRN Journal.

[143] Yoshizawa M, Rafeedie J, Tang JJ, Lei BT, Durazo-Arvizu R, Azucar D, Hudson S, Rao S, Imagawa KK, Deavenport-Saman A (2026). Screen time, child depression, and anxiety during the COVID-19 pandemic: systematic review and meta-analysis. JMIR Pediatr Parent.

[144] Han X, Zhou E, Liu D (2024). Electronic media use and sleep quality: updated systematic review and meta-analysis. J Med Internet Res.

[145] Wang J, Li M, Zhu D, Cao Y (2020). Smartphone overuse and visual impairment in children and young adults: systematic review and meta-analysis. J Med Internet Res.

[146] Biddle SJ, García Bengoechea E, Pedisic Z, Bennie J, Vergeer I, Wiesner G (2017). Screen time, other sedentary behaviours, and obesity risk in adults: a review of reviews. Curr Obes Rep.

[147] Kim S, Favotto L, Halladay J, Wang L, Boyle MH, Georgiades K (2020). Differential associations between passive and active forms of screen time and adolescent mood and anxiety disorders. Soc Psychiatry Psychiatr Epidemiol.

