# Supplementary material for: Association of Screen Time With Physical and Mental Health in University Students: Systematic Review and Meta-Analysis
Source: J Med Internet Res. 2026 Oct 26;28:e93848. doi: 10.2196/93848 (PMC13749025; doi:10.2196/93848)
Supplement: Supplementary file 1 [file jmir_v28i1e93848_app1.pdf]

PRISMA 2020 Checklist

Section and Topic	Item #	Checklist item	Location where item is reported
**TITLE**			
Title	1	Identify the report as a systematic review.	Page 1, lines 1-4
**ABSTRACT**			
Abstract	2	See the PRISMA 2020 for Abstracts checklist.	Page 1-2, lines 6-54
**INTRODUCTION**			
Rationale	3	Describe the rationale for the review in the context of existing knowledge.	Page 3-4, lines 82-103
Objectives	4	Provide an explicit statement of the objective(s) or question(s) the review addresses.	Page 4, lines 104-113
**METHODS**			
Eligibility criteria	5	Specify the inclusion and exclusion criteria for the review and how studies were grouped for the syntheses.	Page 6, lines 170-186; Multimedia Appendix 2
Information sources	6	Specify all databases, registers, websites, organisations, reference lists and other sources searched or consulted to identify studies. Specify the date when each source was last searched or consulted.	Page 5-6, lines 132-138; 147-150
Search strategy	7	Present the full search strategies for all databases, registers and websites, including any filters and limits used.	Page 5-6, lines 138-146; 150-

			154, 160-163; Multimedia Appendix 1
Selection process	8	Specify the methods used to decide whether a study met the inclusion criteria of the review, including how many reviewers screened each record and each report retrieved, whether they worked independently, and if applicable, details of automation tools used in the process.	Page 7, lines 183-191
Data collection process	9	Specify the methods used to collect data from reports, including how many reviewers collected data from each report, whether they worked independently, any processes for obtaining or confirming data from study investigators, and if applicable, details of automation tools used in the process.	Page 7, lines 198-201
Data items	10a	List and define all outcomes for which data were sought. Specify whether all results that were compatible with each outcome domain in each study were sought (e.g. for all measures, time points, analyses), and if not, the methods used to decide which results to collect.	Page 7-8, lines 201-211, 225-238; Multimedia Appendix 3
	10b	List and define all other variables for which data were sought (e.g. participant and intervention characteristics, funding sources). Describe any assumptions made about any missing or unclear information.	Page 7-8, lines 201-224
Study risk of bias assessment	11	Specify the methods used to assess risk of bias in the included studies, including details of the tool(s) used, how many reviewers assessed each study and whether they worked independently, and if applicable, details of automation tools used in the process.	Page 8, lines 239-252; Multimedia Appendix 4
Effect measures	12	Specify for each outcome the effect measure(s) (e.g. risk ratio, mean difference) used in the synthesis or presentation of results.	Page 9-10, lines 294-305, 321-327
Synthesis methods	13a	Describe the processes used to decide which studies were eligible for each synthesis (e.g. tabulating the study intervention characteristics and comparing against the planned groups for each synthesis (item #5)).	Page 9-10, lines 274-297

	13b	Describe any methods required to prepare the data for presentation or synthesis, such as handling of missing summary statistics, or data conversions.	Page 10, lines 299-307
	13c	Describe any methods used to tabulate or visually display results of individual studies and syntheses.	Page 10, lines 330-332
	13d	Describe any methods used to synthesize results and provide a rationale for the choice(s). If meta-analysis was performed, describe the model(s), method(s) to identify the presence and extent of statistical heterogeneity, and software package(s) used.	Page 10-11, lines 308-341
	13e	Describe any methods used to explore possible causes of heterogeneity among study results (e.g. subgroup analysis, meta-regression).	Page 11-12, lines 337-341, 355-369
	13f	Describe any sensitivity analyses conducted to assess robustness of the synthesized results.	Page 11, lines 347-354
Reporting bias assessment	14	Describe any methods used to assess risk of bias due to missing results in a synthesis (arising from reporting biases).	Page 11, lines 342-344
Certainty assessment	15	Describe any methods used to assess certainty (or confidence) in the body of evidence for an outcome.	Page 8-9, lines 253-272; Multimedia Appendix 5
**RESULTS**			
Study selection	16a	Describe the results of the search and selection process, from the number of records identified in the search to the number of studies included in the review, ideally using a flow diagram.	Page 13, lines 373-388; Figure 1
	16b	Cite studies that might appear to meet the inclusion criteria, but which were excluded, and explain why they were excluded.	Page 13, lines 389-391;

			Multimedia Appendix 2
Study characteristics	17	Cite each included study and present its characteristics.	Page 14-15, lines 395-427; Multimedia Appendix 3
Risk of bias in studies	18	Present assessments of risk of bias for each included study.	Page 15, lines 429-439; Multimedia Appendix 4
Results of individual studies	19	For all outcomes, present, for each study: (a) summary statistics for each group (where appropriate) and (b) an effect estimate and its precision (e.g. confidence/credible interval), ideally using structured tables or plots.	Page 16, 456-462; Figure 2-5
Results of syntheses	20a	For each synthesis, briefly summarise the characteristics and risk of bias among contributing studies.	Page 16-24, lines 456-553; Figure 2-5 Multimedia Appendix 4
	20b	Present results of all statistical syntheses conducted. If meta-analysis was done, present for each the summary estimate and its precision (e.g. confidence/credible interval) and measures of statistical heterogeneity. If comparing groups, describe the direction of the effect.	Page 16-22, lines 456-462, 490-502, 509-528, 535-553; Figure 2-5
	20c	Present results of all investigations of possible causes of heterogeneity among study results.	Page18-22, lines 489-553; Page 23, lines, 560-572; Figure

			3-5; Table 2
	20d	Present results of all sensitivity analyses conducted to assess the robustness of the synthesized results.	Page 17, lines 467-488; Table 1; Multimedia Appendix 7
Reporting biases	21	Present assessments of risk of bias due to missing results (arising from reporting biases) for each synthesis assessed.	Page 17, lines 467-475; Multimedia Appendix 5
Certainty of evidence	22	Present assessments of certainty (or confidence) in the body of evidence for each outcome assessed.	Page 15-16, lines 441-453; Multimedia Appendix 5
**DISCUSSION**			
Discussion	23a	Provide a general interpretation of the results in the context of other evidence.	Page 25-28, lines 578-682
	23b	Discuss any limitations of the evidence included in the review.	Page 28-29, lines 699-711,716-718
	23c	Discuss any limitations of the review processes used.	Page 29, lines 712-718
	23d	Discuss implications of the results for practice, policy, and future research.	Page 29-30, lines 734-745

OTHER INFORMATION			
Registration and protocol	24a	Provide registration information for the review, including register name and registration number, or state that the review was not registered.	Page1, lines 55
	24b	Indicate where the review protocol can be accessed, or state that a protocol was not prepared.	Page 1, lines 56
	24c	Describe and explain any amendments to information provided at registration or in the protocol.	Page 5, lines 122-130
Support	25	Describe sources of financial or non-financial support for the review, and the role of the funders or sponsors in the review.	Page 31, lines 759-762, 777
Competing interests	26	Declare any competing interests of review authors.	Page 32, lines 787
Availability of data, code and other materials	27	Report which of the following are publicly available and where they can be found: template data collection forms; data extracted from included studies; data used for all analyses; analytic code; any other materials used in the review.	Multimedia Appendix 1-7

From: Page MJ, McKenzie JE, Bossuyt PM, Boutron I, Hoffmann TC, Mulrow CD, et al. The PRISMA 2020 statement: an updated guideline for reporting systematic reviews. BMJ 2021;372:n71. doi: 10.1136/bmj.n71. This work is licensed under CC BY 4.0. To view a copy of this license, visit https://creativecommons.org/licenses/by/4.0/
