# Supplementary material for: Association of Screen Time With Physical and Mental Health in University Students: Systematic Review and Meta-Analysis
Source: J Med Internet Res. 2026 Oct 26;28:e93848. doi: 10.2196/93848 (PMC13749025; doi:10.2196/93848)
Supplement: Supplementary file 3 [file jmir_v28i1e93848_app2.docx]

**Table S1: Search Strategy**

| **PubMed (1967 to 11 July 2026)** | | | |
| --- | --- | --- | --- |
| **Search number** | **Query** | **Filters** | **Results** |
| **1** | (((((((((((((("Body Composition"[Mesh]) OR "Body Fat Distribution"[Majr]) OR "Adiposity"[Majr]) OR "Obesity"[Mesh]) OR "Insulin Resistance"[Mesh]) OR "Metabolic Syndrome"[Mesh]) OR "Diabetes Mellitus"[Mesh]) OR "Heart Diseases"[Mesh]) OR "Hypertension"[Mesh]) OR "Mental Health"[Mesh]) OR "Sleep"[Mesh]) OR "Diet"[Mesh]) OR "Dry Eye Syndromes"[Majr]) OR ("Body composition"[Title/Abstract] OR "Body fat"[Title/Abstract] OR "Obes*"[Title/Abstract] OR "Overweight"[Title/Abstract] OR "Body Mass Index"[Title/Abstract] OR "BMI"[Title/Abstract] OR "Weight"[Title/Abstract] OR "Diabet*"[Title/Abstract] OR "diabetes mellitus"[Title/Abstract] OR "Type 2 diabetes"[Title/Abstract] OR "Type 2 diabetes mellitus"[Title/Abstract] OR "Insulin Resistance"[Title/Abstract] OR "Insulin Sensitivity"[Title/Abstract] OR "Insulin Sensitivity Index"[Title/Abstract] OR "Glucose intolerance"[Title/Abstract] OR "Hyperinsulinemia"[Title/Abstract] OR "Insulin receptor dysfunction"[Title/Abstract] OR "Pre-diabetes"[Title/Abstract] OR "Metabolic syndrome"[Title/Abstract] OR "Cardiometabolic syndrome"[Title/Abstract] OR "Dyslipidaemia"[Title/Abstract] OR "Hyperglyc*"[Title/Abstract] OR "Abdominal obesity"[Title/Abstract] OR "Cardiovascular disease*"[Title/Abstract] OR "CVD"[Title/Abstract] OR "Cardiovascular disorder*"[Title/Abstract] OR "Heart disease*"[Title/Abstract] OR "Coronary Artery Disease*"[Title/Abstract] OR "CAD"[Title/Abstract] OR "Coronary Heart Disease*"[Title/Abstract] OR "CHD"[Title/Abstract] OR "Hyperten*"[Title/Abstract] OR "Stroke"[Title/Abstract] OR "Peripheral Arterial Disease*"[Title/Abstract] OR "Atherosclerosis"[Title/Abstract] OR "Heart failure"[Title/Abstract] OR "Arrhythmias"[Title/Abstract] OR "Cardiomyopathy"[Title/Abstract] OR "Hyperlipidaemia"[Title/Abstract] OR "Blood pressure"[Title/Abstract] OR "Cholesterol level*"[Title/Abstract] OR "Heart disease risk factors"[Title/Abstract] OR "Hypertensive disease"[Title/Abstract] OR "Mental health"[Title/Abstract] OR "Anxiety"[Title/Abstract] OR "Depress*"[Title/Abstract] OR "Wellbeing"[Title/Abstract] OR "Well-being"[Title/Abstract] OR "Quality of life"[Title/Abstract] OR "Health-related quality of life"[Title/Abstract] OR "HRQoL"[Title/Abstract] OR "Sleep"[Title/Abstract] OR "Sleep hygiene"[Title/Abstract] OR "Sleep disorder"[Title/Abstract] OR "Diet*"[Title/Abstract] OR "Eating beha*"[Title/Abstract] OR "Dry eye syndrome*"[Title/Abstract] OR "Eye health"[Title/Abstract] OR "Eye disease*" [Title/Abstract] OR "Eye disorder"[Title/Abstract] OR "Dry eye*"[Title/Abstract])) AND (((((((((((((((("Screen Time"[Majr])) OR "Mobile Applications"[Majr]) OR "Online Social Networking"[Majr]) OR "Internet Use"[Majr]) OR "Multimedia"[Majr]) OR "Smartphone"[Mesh]) OR "Social Media"[Mesh]) OR "Software"[Mesh]) OR "Telephone"[Mesh]) OR "Virtual Reality"[Mesh]) OR "Computers"[Mesh]) OR "Digital Technology"[Majr]) OR "Electronic Mail"[Majr]) OR (("Screen Time"[Title/Abstract] OR "Screen view*"[Title/Abstract] OR "Television view"[Title/Abstract] OR "TV"[Title/Abstract] OR "Computer"[Title/Abstract] OR "Laptop"[Title/Abstract] OR "Tablet use*"[Title/Abstract] OR "MacBook"[Title/Abstract] OR "Internet use*"[Title/Abstract] OR "Media consumption"[Title/Abstract] OR "Smartphone*"[Title/Abstract] OR "Cell phone"[Title/Abstract] OR "Video game*"[Title/Abstract] OR "Electronic game*"[Title/Abstract] OR "Electronic device use*"[Title/Abstract] OR "Social media*"[Title/Abstract] OR "Social network*"[Title/Abstract] OR "Text Message"[Title/Abstract] OR "Mobile device*"[Title/Abstract] OR "Digital media*"[Title/Abstract] OR "Online"[Title/Abstract] OR "Digital"[Title/Abstract] OR "DVD"[Title/Abstract] OR "Electronic"[Title/Abstract] OR "Email*"[Title/Abstract] OR "Internet"[Title/Abstract] OR "Mobile"[Title/Abstract] OR "Multimedia"[Title/Abstract] OR "SMS"[Title/Abstract] OR "Software"[Title/Abstract] OR "Video"[Title/Abstract] OR "Virtual"[Title/Abstract] OR "Webs*"[Title/Abstract]))) AND ((("Universities"[Mesh])) OR (("University student*"[Title/Abstract] OR "College student*"[Title/Abstract] OR "Undergraduate*"[Title/Abstract] OR "Postgraduate student*"[Title/Abstract] OR "Postgraduate*"[Title/Abstract] OR "Higher education"[Title/Abstract] OR "Higher education institution*"[Title/Abstract] OR "HEI"[Title/Abstract])))) | English, full text, from database inception through 2026/07/11 | 12,735 |

| **APA PsycInfo (via EBSCO host Research)** | | | |
| --- | --- | --- | --- |
| **Search** | **Query (user-entered)** | **Results (count)** | **filters** |
| **S1** | [MA (“Screen time” OR "Mobile applications" OR "Online social networking" OR "Internet use" OR "Multimedia" OR "Smartphone" OR "Social media" OR "Software" OR "Telephone" OR "Virtual reality" OR "Computer" OR "Digital technology" OR "Electronic mail") OR XB (“Screen time” OR “Screen view*” OR “Television view” OR “TV” OR “Computer” OR “Laptop” OR “Tablet use*” OR "MacBook" OR "Internet use*" OR "Media consumption" OR "Smartphone*" OR "Cell phone*" OR "Video game*" OR "Electronic game*" OR "Electronic device use*" OR "Social media*" OR "Social network*" OR "Text Message*" OR "Mobile device*" OR "Digital media*" OR "Online" OR "Digital" OR "DVD" OR "Electronic" OR "Email*" OR "Internet" OR "Mobile" OR "Multimedia" OR "SMS" OR "Software" OR "Video" OR "Virtual" OR "Webs*")](https://research.ebsco.com/c/v64bvo/search/advanced-results?db=psyh&expanders=concept&limiters=None&q=MA%20%28%E2%80%9CScreen%20time%E2%80%9D%20OR%20%22Mobile%20applications%22%20OR%20%22Online%20social%20networking%22%20OR%20%22Internet%20use%22%20OR%20%22Multimedia%22%20OR%20%22Smartphone%22%20OR%20%22Social%20media%22%20OR%20%22Software%22%20OR%20%22Telephone%22%20OR%20%22Virtual%20reality%22%20OR%20%22Computer%22%20OR%20%22Digital%20technology%22%20OR%20%22Electronic%20mail%22%29%20OR%20XB%20%28%E2%80%9CScreen%20time%E2%80%9D%20OR%20%E2%80%9CScreen%20view%2A%E2%80%9D%20OR%20%E2%80%9CTelevision%20view%E2%80%9D%20OR%20%E2%80%9CTV%E2%80%9D%20OR%20%E2%80%9CComputer%E2%80%9D%20OR%20%E2%80%9CLaptop%E2%80%9D%20OR%20%E2%80%9CTablet%20use%2A%E2%80%9D%20OR%20%22MacBook%22%20OR%20%22Internet%20use%2A%22%20OR%20%22Media%20consumption%22%20OR%20%22Smartphone%2A%22%20OR%20%22Cell%20phone%2A%22%20OR%20%22Video%20game%2A%22%20OR%20%22Electronic%20game%2A%22%20OR%20%22Electronic%20device%20use%2A%22%20OR%20%22Social%20media%2A%22%20OR%20%22Social%20network%2A%22%20OR%20%22Text%20Message%2A%22%20OR%20%22Mobile%20device%2A%22%20OR%20%22Digital%20media%2A%22%20OR%20%22Online%22%20OR%20%22Digital%22%20OR%20%22DVD%22%20OR%20%22Electronic%22%20OR%20%22Email%2A%22%20OR%20%22Internet%22%20OR%20%22Mobile%22%20OR%20%22Multimedia%22%20OR%20%22SMS%22%20OR%20%22Software%22%20OR%20%22Video%22%20OR%20%22Virtual%22%20OR%20%22Webs%2A%22%29&searchMode=all&searchQueryId=sq%3A39b76988-4746-48ff-ac79-bd8b3e0c5042&sort=relevance&userDirectAction=true) | 580,583 |  |
| **S2** | [MA ("Universities") OR XB ("University student*" OR "College student*" OR "Undergraduate*" OR "Postgraduate student*" OR "Postgraduate*" OR "Higher education" OR ("Higher education institution*" OR "HEI")](https://research.ebsco.com/c/v64bvo/search/advanced-results?db=psyh&expanders=concept&limiters=None&q=MA%20%28%22Universities%22%29%20OR%20XB%20%28%22University%20student%2A%22%20OR%20%22College%20student%2A%22%20OR%20%22Undergraduate%2A%22%20OR%20%22Postgraduate%20student%2A%22%20OR%20%22Postgraduate%2A%22%20OR%20%22Higher%20education%22%20OR%20%28%22Higher%20education%20institution%2A%22%20OR%20%22HEI%22%29&searchMode=all&searchQueryId=sq%3A2590e605-e72f-4c55-a590-1057160eadae&sort=relevance&userDirectAction=true) | 376,669 |  |
| **S3** | [(S1) AND (S2)](https://research.ebsco.com/c/v64bvo/search/advanced-results?combinedSearchQueryId=sq%3Afaf09761-35e8-4a08-9608-b207d49b9b9d&db=psyh&expanders=concept&limiters=None&q=%28S1%29%20AND%20%28S2%29&searchMode=all&searchQueryId=sq%3Afaf09761-35e8-4a08-9608-b207d49b9b9d&sort=relevance&userDirectAction=true) | 52,185 |  |
| **S4** | [MA ("Body composition" OR "Body Fat Distribution" OR "Adiposity" OR "Insulin Resistance" OR "Metabolic Syndrome" OR "Diabetes Mellitus" OR "Heart Disease Risk Factors" OR "Hypertension" OR "Mental Health" OR "Sleep" OR "Diet" OR "Dry Eye Syndromes") OR XB ("Body composition" OR "Body fat" OR "Obes*" OR "Overweight” OR "Body Mass Index" OR "BMI" OR "Weight" OR "Diabet*" OR "diabetes mellitus" OR "Type 2 diabetes” OR "Type 2 diabetes mellitus" OR "Insulin Resistance" OR "Insulin Sensitivity" OR "Insulin Sensitivity Index" OR "Glucose intolerance" OR "Hyperinsulinemia" OR "Insulin receptor dysfunction" OR "Pre-diabetes" OR "Metabolic syndrome" OR "Cardiometabolic syndrome" OR "Dyslipidaemia" OR "Hyperglyc*" OR "Abdominal obesity" OR "Cardiovascular disease*" OR "CVD" OR ("Cardiovascular disorder*" OR "Heart disease*" OR "Coronary Artery Disease*" OR "CAD" OR "Coronary Heart Disease*" OR "CHD" OR "Hyperten*" OR "Stroke" OR "Peripheral Arterial Disease*" OR "Atherosclerosis" OR "Heart failure" OR "Arrhythmias" OR "Cardiomyopathy" OR "Hyperlipidaemia" OR "Blood pressure" OR "Cholesterol level*" OR "Heart disease risk factors" OR "Hypertensive disease" OR "Mental health" OR "Anxiety" OR "Depress*" OR "Wellbeing" OR "Well-being" OR "Quality of life" OR "Health-related quality of life" OR "HRQoL" OR "Sleep" OR "Sleep hygiene" OR "Sleep disorder" OR "Diet*" OR "Eating beha*" OR ("Dry eye syndrome*" OR "Eye health" OR "Eye disease*"OR "Eye disorder" OR "Dry eye*")](https://research.ebsco.com/c/v64bvo/search/advanced-results?db=psyh&expanders=concept&limiters=None&q=MA%20%28%22Body%20composition%22%20OR%20%22Body%20Fat%20Distribution%22%20OR%20%22Adiposity%22%20OR%20%22Insulin%20Resistance%22%20OR%20%22Metabolic%20Syndrome%22%20OR%20%22Diabetes%20Mellitus%22%20OR%20%22Heart%20Disease%20Risk%20Factors%22%20OR%20%22Hypertension%22%20OR%20%22Mental%20Health%22%20OR%20%22Sleep%22%20OR%20%22Diet%22%20OR%20%22Dry%20Eye%20Syndromes%22%29%20OR%20XB%20%28%22Body%20composition%22%20OR%20%22Body%20fat%22%20OR%20%22Obes%2A%22%20OR%20%22Overweight%E2%80%9D%20OR%20%22Body%20Mass%20Index%22%20OR%20%22BMI%22%20OR%20%22Weight%22%20OR%20%22Diabet%2A%22%20OR%20%22diabetes%20mellitus%22%20OR%20%22Type%202%20diabetes%E2%80%9D%20OR%20%22Type%202%20diabetes%20mellitus%22%20OR%20%22Insulin%20Resistance%22%20OR%20%22Insulin%20Sensitivity%22%20OR%20%22Insulin%20Sensitivity%20Index%22%20OR%20%22Glucose%20intolerance%22%20OR%20%22Hyperinsulinemia%22%20OR%20%22Insulin%20receptor%20dysfunction%22%20OR%20%22Pre-diabetes%22%20OR%20%22Metabolic%20syndrome%22%20OR%20%22Cardiometabolic%20syndrome%22%20OR%20%22Dyslipidaemia%22%20OR%20%22Hyperglyc%2A%22%20OR%20%22Abdominal%20obesity%22%20OR%20%22Cardiovascular%20disease%2A%22%20OR%20%22CVD%22%20OR%20%28%22Cardiovascular%20disorder%2A%22%20OR%20%22Heart%20disease%2A%22%20OR%20%22Coronary%20Artery%20Disease%2A%22%20OR%20%22CAD%22%20OR%20%22Coronary%20Heart%20Disease%2A%22%20OR%20%22CHD%22%20OR%20%22Hyperten%2A%22%20OR%20%22Stroke%22%20OR%20%22Peripheral%20Arterial%20Disease%2A%22%20OR%20%22Atherosclerosis%22%20OR%20%22Heart%20failure%22%20OR%20%22Arrhythmias%22%20OR%20%22Cardiomyopathy%22%20OR%20%22Hyperlipidaemia%22%20OR%20%22Blood%20pressure%22%20OR%20%22Cholesterol%20level%2A%22%20OR%20%22Heart%20disease%20risk%20factors%22%20OR%20%22Hypertensive%20disease%22%20OR%20%22Mental%20health%22%20OR%20%22Anxiety%22%20OR%20%22Depress%2A%22%20OR%20%22Wellbeing%22%20OR%20%22Well-being%22%20OR%20%22Quality%20of%20life%22%20OR%20%22Health-related%20quality%20of%20life%22%20OR%20%22HRQoL%22%20OR%20%22Sleep%22%20OR%20%22Sleep%20hygiene%22%20OR%20%22Sleep%20disorder%22%20OR%20%22Diet%2A%22%20OR%20%22Eating%20beha%2A%22%20OR%20%28%22Dry%20eye%20syndrome%2A%22%20OR%20%22Eye%20health%22%20OR%20%22Eye%20disease%2A%22OR%20%22Eye%20disorder%22%20OR%20%22Dry%20eye%2A%22%29&searchMode=all&searchQueryId=sq%3A487fda4c-3799-4126-923c-5f49e5be05c9&sort=relevance&userDirectAction=true) | 1,831,982 |  |
| **S5** | [(S4) AND (S3)](https://research.ebsco.com/c/v64bvo/search/advanced-results?combinedSearchQueryId=sq%3Afc7d19c7-d5f6-47d4-b3ce-eff5b71eb519&db=psyh&expanders=concept&limiters=None&q=%28S4%29%20AND%20%28S3%29&searchMode=all&searchQueryId=sq%3Afc7d19c7-d5f6-47d4-b3ce-eff5b71eb519&sort=relevance&userDirectAction=true) | 7,276 | From database inception to 07/2026; Linked Full Text; English; Peer Reviewed |

| **CINAHL Ultimate (via EBSCO host Research)** | | | |
| --- | --- | --- | --- |
| **Search** | **Query (user-entered)** | **Results (count)** | **Filters** |
| **S1** | MH ("Screen time" OR "Mobile applications" OR "Online social networking" OR "Internet use" OR "Multimedia" OR "Smartphone" OR "Social media" OR "Software" OR "Telephone" OR "Virtual reality" OR "Computer" OR "Digital technology" OR "Electronic mail") OR XB ((“Screen time” OR “Screen view*” OR “Television view” OR “TV” OR “Computer” OR “Laptop” OR “Tablet use*” OR "MacBook" OR "Internet use*" OR "Media consumption" OR "Smartphone*" OR "Cell phone*" OR "Video game*" OR "Electronic game*" OR "Electronic device use*" OR "Social media*" OR "Social network*" OR "Text Message*" OR "Mobile device*" OR "Digital media*" OR "Online" OR "Digital" OR "DVD" OR "Electronic" OR "Email*" OR "Internet" OR "Mobile" OR "Multimedia" OR "SMS" OR "Software" OR "Video" OR "Virtual" OR "Webs*")) | 623,878 |  |
| **S2** | MH ("Universities") OR XB (("University student*" OR "College student*" OR "Undergraduate*" OR "Postgraduate student*" OR "Postgraduate*" OR "Higher education" OR ("Higher education institution*" OR "HEI")) | 81,089 |  |
| **S3** | (S2) AND (S1) | 17,666 |  |
| **S4** | MH (("Body composition" OR "Body Fat Distribution" OR "Adiposity" OR "Insulin Resistance" OR "Metabolic Syndrome" OR "Diabetes Mellitus" OR "Heart Disease Risk Factors" OR "Hypertension" OR "Mental Health" OR "Sleep" OR "Diet" OR "Dry Eye Syndromes")) OR XB (("Body composition" OR "Body fat" OR "Obes*" OR "Overweight” OR "Body Mass Index" OR "BMI" OR "Weight" OR "Diabet*" OR "diabetes mellitus" OR "Type 2 diabetes” OR "Type 2 diabetes mellitus" OR "Insulin Resistance" OR "Insulin Sensitivity" OR "Insulin Sensitivity Index" OR "Glucose intolerance" OR "Hyperinsulinemia" OR "Insulin receptor dysfunction" OR "Pre-diabetes" OR "Metabolic syndrome" OR "Cardiometabolic syndrome" OR "Dyslipidemia" OR "Hyperglyc*" OR "Abdominal obesity" OR "Cardiovascular disease*" OR "CVD" OR ("Cardiovascular disorder*" OR "Heart disease*" OR "Coronary Artery Disease*" OR "CAD" OR "Coronary Heart Disease*" OR "CHD" OR "Hyperten*" OR "Stroke" OR "Peripheral Arterial Disease*" OR "Atherosclerosis" OR "Heart failure" OR "Arrhythmias" OR "Cardiomyopathy" OR "Hyperlipidemia" OR "Blood pressure" OR "Cholesterol level*" OR "Heart disease risk factors" OR "Hypertensive disease" OR "Mental health" OR "Anxiety" OR "Depress*" OR "Wellbeing" OR "Well-being" OR "Quality of life" OR "Health-related quality of life" OR "HRQoL" OR "Sleep" OR "Sleep hygiene" OR "Sleep disorder" OR "Diet*" OR "Eating beha*" OR ("Dry eye syndrome*" OR "Eye health" OR "Eye disease*"OR "Eye disorder" OR "Dry eye*")) | 2,214,674 |  |
| **S5** | (S4) AND (S3) | 4,208 | Date: from database inception to 07/2026; Full text; English; Peer Reviewed |

| **Web of Science Core Collection** | | | |
| --- | --- | --- | --- |
| **Search** | **Query (user-entered)** | **Results (count)** | **Filters** |
| **1** | "University student*" OR "College student*" OR "Undergraduate*" OR "Postgraduate*" (Title) or "University student*" OR "College student*" OR "Undergraduate*" OR "Postgraduate*" (Abstract) | 362,518 |  |
| **2** | "Screen time" OR "screen use" OR "Screen exposure" OR "smartphone*" OR "Mobile phone*" OR "Television" OR "TV" (Title) or "Screen time" OR "screen use" OR "Screen exposure" OR "smartphone*" OR "Mobile phone*" OR "Television" OR "TV" (Abstract) | 282,750 |  |
| **3** | #1 AND #2 | 5,629 |  |
| **4** | "Sleep" OR "Depression" OR "Anxiety" OR "Stress" OR "Wellbeing" OR "Quality of life" OR "HRQoL" OR "Obesity" OR "Overweight" OR "BMI" OR "Dry eye"(Title) or “Sleep" OR "Depression" OR "Anxiety" OR "Stress" OR "Wellbeing" OR "Quality of life" OR "HRQoL" OR "Obesity" OR "Overweight" OR "BMI" OR "Dry eye" (Abstract) | 4,517,844 |  |
| **5** | #3 AND #4 | 1,381 | English; Article |
