# Supplementary material for: Association of Screen Time With Physical and Mental Health in University Students: Systematic Review and Meta-Analysis
Source: J Med Internet Res. 2026 Oct 26;28:e93848. doi: 10.2196/93848 (PMC13749025; doi:10.2196/93848)
Supplement: Supplementary file 4 [file jmir_v28i1e93848_app4.docx]

**Table S2. Inclusion and Exclusion Criteria**

**Inclusion Criteria**

1. Studies including university students aged ≥17 years or studies in which data for eligible university students could be extracted separately

2. Screen time assessed as duration (hours and/or minutes) using objective methods (eg, device-based measures or accelerometry) or self-reported measures.

3. Measure of health indicators among university students

•	Body composition and weight-related outcomes: BMI/weight gain/food dietary behavior

•	Metabolic syndrome and cardiovascular disease factors, including blood pressure, lipid profiles, or markers of insulin resistance or type 2 diabetes

•	Sleep related outcomes: particularly sleep quality measured using validated instruments (eg, the Pittsburgh Sleep Quality Index [PSQI])

•	Eye health, including dry eye symptoms or eye strain

- Mental health: anxiety, depression, stress, psychological distress, or overall well-being assessed using validated instruments

4. Observational studies or experimental studies with baseline data for both screen time and health outcomes

5. Statistical data that can be extracted and transformed into an effect size (eg, correlation, odds ratio, standardized regression coefficients, means and standard deviations, etc.)

6. Articles published in peer-reviewed journals in English

7. Minimum sample size thresholds defined a priori:

•	Observational studies: ≥300 participants

- Randomized controlled trials: ≥30 participants

**Exclusion Criteria**

1. Studies including part-time university students or participants aged < 17 years

2. Studies focusing exclusively on clinical populations (eg, pregnancy or disability related conditions)

3. No measure of university students’ screen time in terms of duration (eg, only frequency of screen use, screen addiction scales, or problematic internet use)

4. Studies examining specific screen content only (eg, violent or educational content) without reporting the duration of screen use.

5. Did not measure health indicators in terms of physical or mental health (eg, temperament, self-esteem/self-regulation, or personality traits only)

6. Screen used as experimental condition (eg, examined behavior after viewing a 30-minute video in a lab setting)

7. Studies without extractable statistical data required for effect size calculation

8. Full text studies published in languages other than English

**Exclusion study**

| Number | Study | Exclusion reason |
| --- | --- | --- |
| 1 | Abdel-Salam et al, 2019 | Internet addiction, without valid measure for sleep quality |
| 2 | Aman et al, 2018 | Internet addition |
| 3 | Aslan & Polat, 2024 | Focus on social media addiction |
| 4 | Benaich et al, 2020 | No validate measurement of sleep quality |
| 5 | Boumosleh et al, 2017 | Smartphone addiction |
| 6 | Abu‐Snieneh et al, 2020 | Social media addiction |
| 7 | Gunjan et al, 2022 | Internet addition |
| 8 | Hana Mohammad, 2020 | Social media addiction |
| 9 | Li et al, 2023 | Game disorder |
| 10 | Zhang et al, 2023 | Although it reported screen time, but the results only test the relationship between smartphone addiction and depression |
| 11 | Soares et al, 2019 | Internet gaming disorder |
| 12 | Tawil et al, 2018 | Didn't directly report the association between screen time and dry eyes. |
| 13 | Lima et al, 2019 | two independent analytic samples whose combined sample size exceeded 300. However, each individual sample contained fewer than 300 participants |

Reference list:

1. Abdel-Salam, D. M., Alrowaili, H. I., Albedaiwi, H. K., Alessa, A. I., & Alfayyadh, H. A. (2019). Prevalence of Internet addiction and its associated factors among female students at Jouf University, Saudi Arabia. *Journal of the Egyptian Public Health Association*, *94*(1), 12.

2. Gupta, A., Khan, A. M., Rajoura, O. P., & Srivastava, S. (2018). Internet addiction and its mental health correlates among undergraduate college students of a university in North India. *Journal of family medicine and primary care*, *7*(4), 721.
3. Aslan, I., & Polat, H. (2024). Investigating social media addiction and impact of social media addiction, loneliness, depression, life satisfaction and problem-solving skills on academic self-efficacy and academic success among university students. *Frontiers in Public Health*, *12*, 1359691.
4. Benaich, S., Mehdad, S., Andaloussi, Z., Boutayeb, S., Alamy, M., Aguenaou, H., & Taghzouti, K. (2021). Weight status, dietary habits, physical activity, screen time and sleep duration among university students. *Nutrition and Health*, *27*(1), 69-78.
5. Matar Boumosleh, J., & Jaalouk, D. (2017). Depression, anxiety, and smartphone addiction in university students-A cross sectional study. *PloS one*, *12*(8), e0182239.
6. Abu‐Snieneh, H. M., Aroury, A. M. D. A., Alsharari, A. F., Al‐Ghabeesh, S. H., & Esaileh, A. A. (2020). Relationship between sleep quality, using social media platforms, and academic performance among university students. *Perspectives in psychiatric care*, *56*(2), 415.
7. Kumar, G., Dash, P., Jnaneswar, A., Suresan, V., Jha, K., & Ghosal, S. (2022). Impact of internet addiction during COVID-19 on anxiety and sleep quality among college students of Bhubaneswar city. *Journal of education and health promotion*, *11*, 156.
8. Abu‐Snieneh, H. M., Aroury, A. M. D. A., Alsharari, A. F., Al‐Ghabeesh, S. H., & Esaileh, A. A. (2020). Relationship between sleep quality, using social media platforms, and academic performance among university students. *Perspectives in psychiatric care*, *56*(2), 415.
9. Li, L., Liu, L., Niu, Z., Zhong, H., Mei, S., & Griffiths, M. D. (2023). Gender differences and left-behind experiences in the relationship between gaming disorder, rumination and sleep quality among a sample of Chinese university students during the late stage of the COVID-19 pandemic. *Frontiers in Psychiatry*, *14*, 1108016.
10. Zhang, K., Guo, H., Wang, T., Zhang, J., Yuan, G., Ren, J., ... & Zhang, Z. (2023). A bidirectional association between smartphone addiction and depression among college students: A cross-lagged panel model. *Frontiers in Public Health*, *11*, 1083856.
11. Severo, R. B., Soares, J. M., Affonso, J. P., Giusti, D. A., de Souza, A. A., de Figueiredo, V. L., ... & Pontes, H. M. (2020). Prevalence and risk factors for internet gaming disorder. *Brazilian Journal of Psychiatry*, *42*(5), 532-535.
12. Al Tawil, L., Aldokhayel, S., Zeitouni, L., Qadoumi, T., Hussein, S., & Ahamed, S. S. (2020). Prevalence of self-reported computer vision syndrome symptoms and its associated factors among university students. *European journal of ophthalmology*, *30*(1), 189-195.
13. Lima, D. V. G., Kluthcovsky, A. C. G. C., Fernandes, L. G. R., & Okarenski, G. (2019). Quality of sleep and use of computers and cell-phones among university students. *Revista da Associação Médica Brasileira*, *65*(12), 1454-1458.
