# Supplementary material for: Association of Screen Time With Physical and Mental Health in University Students: Systematic Review and Meta-Analysis
Source: J Med Internet Res. 2026 Oct 26;28:e93848. doi: 10.2196/93848 (PMC13749025; doi:10.2196/93848)
Supplement: Supplementary file 5 [file jmir_v28i1e93848_app5.docx]

**Table S3. Characteristics of Included Studies**

| **First author (Year)** | **N (Sample size)** | **Health type** | **Exposure** | **Study design** | **Geographic** | **Mental health outcome** | **Physical health outcome** | **Outcome measure/instrument** | **Female (%)** | **Correlation (r)** | **Publication year** | **Study quality** | **Mean age** |
| --- | --- | --- | --- | --- | --- | --- | --- | --- | --- | --- | --- | --- | --- |
| Achak et al, 2023 | 2,419 | Mental | TST | Cross-sectional | Others | Well-being | . | The French version of the EQ-5D-5L instrument | 78.2 | 0.19 | 2023 | 5 | . |
| Akca et al. 2019 | 1,040 | Physical | Smartphone | Cross-sectional | Asia | . | Sleep quality | PSQI | 61.2 | 2.64E-01 | 2019 | 3 | 21.1 |
| Akcay D & Akcay B, 2020 | 892 | Physical | RST | Cross-sectional | Europe | . | Sleep quality | PSQI | 70.5 | 1.08E-01 | 2020 | 7 | 22.76 |
| Akcay et al. 2020 | 752 | Physical | TST | Cross-sectional | Europe | . | Sleep quality | PSQI | 56.5 | 9.80E-02 | 2021 | 6 | 20.57 |
| Akowuah et al, 2021 | 362 | Physical | TST | Cross-sectional | Others | . | Sleep quality | PSQI | 54.97 | 1.47E-01 | 2021 | 8 | 20.96 |
| Al-Amin et al, 2025 | 410 | Mental | TST | Cross-sectional | Asia | Depression | . | PHQ-9 | 49 | 2.14E-01 | 2025 | 9 | 23.1 |
| Alashqar et al, 2025 | 426 | Physical | TST | Cross-sectional | Others | . | Eye health | OSDI | 58.5 | 1.30E-01 | 2025 | 7 | 20.4 |
| Aljaberi et al, 2025 | 1,154 | Physical | TST | Cross-sectional | Others | . | Eye health | OSDI | 63.6 | 2.18E-01 | 2025 | 6 | . |
| Allwihan et al, 2024 | 359 | Physical | TST | Cross-sectional | Others | . | Eye health | CVS-Q | 70.8 | 1.10E-01 | 2024 | 8 | . |
| Almalki et al, 2026 | 1,564 | Physical | Smartphone | Cross-sectional | Others | . | Sleep quality | PSQI | 83.7 | 6.09E-02 | 2026 | 7 | . |
| Almeida et al, 2022 | 525 | Physical | TSM | Cross-sectional | Europe | . | Sleep quality | BaSIQS | 15.8 | 0.22 | 2022 | 5 | 22.39 |
| Alqurashi et al, 2024 | 457 | Physical | TST | Cross-sectional | Others | . | Eye health | OSDI | 49.9 | 8.95E-02 | 2024 | 5 | 22.2 |
| Amez et al, 2020 | 1,889 | Physical | Smartphone | Cross-sectional | Europe | . | Sleep quality | PSQI | 52.7 | -3.00E-02 | 2020 | 6 | 18.79 |
| Anuradha et al, 2022 | 367 | Physical | Smartphone | Cross-sectional | Asia | . | Sleep quality | PSQI | 60.2 | 1.80E-01 | 2022 | 7 | 19.75 |
| Aridi et al, 2026 | 654 | Physical | TST | Cross-sectional | Others | . | Eye health | OSDI | 54.1 | 6.94E-01 | 2026 | 6 | . |
| Aschale et al, 2024 | 398 | Physical | Smartphone | Cross-sectional | Others | . | Sleep quality | a seven-item self-report tool used to evaluate the type, severity, and effects of insomnia | 45.7 | 0.445801341 | 2024 | 9 | 22.22 |
| Balpande et al, 2024 | 392 | Mental | TSM | Cross-sectional | Asia | Depression | . | DASS-21 | 50.26 | 3.50E-01 | 2024 | 7 | . |
| Barry et al, 2022 | 1,120 | Physical | TSM | Cross-sectional | North America | . | Sleep quality | ASSQ | 56.4 | 6.00E-02 | 2022 | 5 | . |
| Benaich et al, 2021 | 438 | Physical | TST | Cross-sectional | Others | . | Weight | BMI | 59.4 | -6.14E-02 | 2021 | 7 | 21 |
| Brar et al, 2026 | 1,562 | Mental | TSM | Longitudinal | North America | Well-being | . | SWEMWBS | 72.7 | 1.42E-01 | 2026 | 9 | 18 |
| Brown et al, 2022 | 513 | Mental | RST | Cross-sectional | North America | Well-being | . | The Kessler Psychological Distress Scale | 67.6 | 1.68E-01 | 2022 | 8 | 20.78 |
| Cartes et al, 2021 | 1,450 | Physical | TST | Cross-sectional | South America | . | Eye health | DEQ-5 | 65 | 1.50E-01 | 2021 | 5 | 21.1 |
| Chandler et al, 2024 | 513 | Mental | TST | Cross-sectional | North America | Depression | . | Single-item self-reported perceived stress measure | 65.4 | 1.68E-01 | 2024 | 6 | 19.7 |
| Chen et al, 2024 | 997 | Mental | TST | Cross-sectional | Asia | Well-being | . | WHO-5 | 59.6 | 1.64E-01 | 2024 | 8 | 20.2 |
| Daniyal et al, 2022 | 400 | Physical | Smartphone | Cross-sectional | Asia | . | Eye health | DES | 55 | 5.77E-01 | 2022 | 5 | 24.45 |
| Deyo et al, 2024 | 372 | Mental | TST | Cross-sectional | North America | Combined （Anxiety + Depression + Stress） | . | PROMMIS Anxiety Short Form 6a-v1.0 Adult; PROMIS Depression short Form 6a-v1.0 Adult; NIH Toolbox Perceived Stress measure | 63.8 | 1.40E-01 | 2024 | 5 | 19.47 |
| Ezinne et al, 2023 | 400 | Physical | TST | Cross-sectional | Asia | . | Eye health | OSDI | 64.8 | 0.422692559 | 2023 | 7 | . |
| Fang et al, 2023 | 780 | Mental | Smartphone | Cross-sectional | Asia | Combined (Anxiety + Depression + Stress) | . | DASS-21 | 61.3 | 1.85E-01 | 2023 | 4 | . |
| Feng et al, 2014 | 1,106 | Mental | TST | Cross-sectional | Asia | Depression | . | SDS | 42.6 | 1.20E-01 | 2014 | 8 | 18.9 |
| Galle et al, 2025 | 1,674 | Physical | TST | Cross-sectional | Europe | . | Sleep quality | PSQI | 71.3 | 1.67E-01 | 2025 | 7 | 24.06 |
| Garcia et al, 2024 | 771 | Physical | TST | Cross-sectional | South America | . | Sleep quality | PSQI | 65.2 | 2.74E-03 | 2024 | 7 | 24.5 |
| Ge et al, 2020 (1) | 392 | Mental | TST | Cross-sectional | Asia | Stress | . | DASS-21 scale | 0 | 6.68E-02 | 2020 | 4 | 19.93 |
| Ge et al, 2020 (2) | 745 | Mental | TST | Cross-sectional | Asia | Stress | . | DASS-21 scale | 65.5 | 1.21E-01 | 2020 | 4 | 19.93 |
| Hjetland et al, 2021 | 49,051 | Physical | TST | Cross-sectional | Europe | . | Sleep quality | DSM-5 | 69.3 | 1.49E-02 | 2021 | 9 | 22 |
| Hosen et al, 2021 | 1,186 | Physical | TSM | Cross-sectional | Asia | . | Sleep quality | Sleep disturbance | 35.2 | 9.54E-02 | 2021 | 7 | 20 |
| Huang et al, 2020 | 439 | Physical | Smartphone | Cross-sectional | Asia | . | Sleep quality | PSQI | 86 | 2.11E-01 | 2020 | 6 | 18.8 |
| Huang et al, 2022 | 321 | Mental | TST | Cross-sectional | Asia | Depression | . | SAS | 55.76 | 0.133 | 2022 | 2 | 19.72 |
| Jakhar et al, 2023 | 352 | Physical | Computer | Cross-sectional | Asia | . | Eye health | DES | 61.1 | 1.64E-01 | 2023 | 4 | 23.33 |
| Kaewpradit et al, 2025 | 446 | Physical | TST | Cross-sectional | Asia | . | Sleep quality | PSQI | 67.9 | 6.57E-02 | 2025 | 9 | 21 |
| Kalirathinam et al, 2019 | 390 | Physical | TST | Cross-sectional | Asia | . | Weight | BMI | 60.5 | 4.65E-02 | 2019 | 5 | 20.17 |
| Kazhali et al, 2021 | 464 | Physical | TSM | Cross-sectional | Asia | . | Sleep quality | PSQI | 50.9 | 2.82E-01 | 2021 | 5 | . |
| Koly et al, 2021 | 400 | Mental | TSM | Cross-sectional | Asia | Depression | . | PHQ-9 | 51.2 | 3.92E-01 | 2021 | 6 | 22.2 |
| Krishnan et al, 2020 | 450 | Physical | Smartphone | Cross-sectional | Asia | . | Sleep quality | PSQI | 60.27 | 3.10E-01 | 2020 | 6 | 19.98 |
| Kumari et al, 2026 | 1,046 | Physical | TST | Cross-sectional | Asia | . | Eye health | CVS-Q | 61.8 | 8.81E-01 | 2026 | 8 | 20 |
| Lavados-Romo et al, 2021 | 726 | Mental | TST | Cross-sectional | South America | Well-being | . | WHOQOL-BREF | 72 | -3.20E-01 | 2021 | 9 | 18.5 |
| Leung et al, 2021 | 343 | Mental | TSM | Cross-sectional | Asia | Depression | . | CES-D | 64.8 | 2.80E-01 | 2021 | 4 | 20.27 |
| Liebig et al, 2023 | 415 | Physical | TST | Cross-sectional | Europe | . | Sleep quality | PSQI | 70.4 | 6.50E-02 | 2023 | 8 | 24.4 |
| Liu et al, 2023 | 1,453 | Mental | TST | Cross-sectional | Asia | Combined (Anxiety+ Depression) | . | SAS /SDS | 54.4 | 0.25 | 2023 | 5 | 21.47 |
| Lu et al, 2023 | 2,014 | Mental | TST | Cross-sectional | Asia | Combined (Anxiety + Depression+ Stress) | . | DASS-21 | 68.9 | 1.60E-01 | 2023 | 8 | 19 |
| Ma et al, 2020 | 5,233 | Mental | TST | Cross-sectional | Asia | Well-being | . | SHMS V1.0 | 64.97 | 9.81E-02 | 2020 | 5 | 21.74 |
| Maqbool et al, 2024 | 415 | Physical | TST | Cross-sectional | Asia | . | Eye health | CVS-Q | 36.4 | -7.54E-02 | 2024 | 5 | 21.3 |
| Meng et al, 2021 | 1,904 | Physical | Smartphone | Cross-sectional | Asia | . | Sleep quality | PSQI | 67 | 3.10E-02 | 2021 | 6 | 20.66 |
| Noel et al, 2022 | 431 | Mental | TST | Cross-sectional | North America | Combined (Depression + Anxiety) | . | CES-D-10; GAD-7 | 65.4 | 3.61E-01 | 2022 | 6 | 21.2 |
| Patni et al, 2026 | 463 | Physical | TSM | Cross-sectional | Asia | . | Eye health | Self-developed item symptoms score | 64.8 | 9.43E-02 | 2026 | 7 | . |
| Peltzer et al, 2014 | 860 | Mental | TSM | Cross-sectional | Asia | Depression | . | CES-D | 72.7 | 0.155484529 | 2014 | 5 | 20.1 |
| Pham et al, 2021 | 369 | Physical | TST | Cross-sectional | Asia | . | Sleep quality | PSQI | 65.5 | 1.62E-01 | 2021 | 6 | 20.3 |
| Rakicioglu N et al, 2026 | 818 | Physical | TST | Cross-sectional | Europe | . | Sleep quality | PSQI | 21.7 | 9.30E-02 | 2026 | 5 | 21.7 |
| Rana et al, 2023 | 497 | Physical | TST | Cross-sectional | Asia | . | Eye health | CVS-Q | 100 | 9.51E-02 | 2023 | 4 | . |
| Rosenthal et al, 2021 | 426 | Mental | Smartphone | Cross-sectional | North America | Depression | . | CES-D10 | 65 | 2.96E-01 | 2021 | 5 | . |
| Sarker et al, 2026 | 683 | Mental | TSM | Cross-sectional | Asia | Combined (Depression + Anxiety + Stress) | . | DASS-21 | 54.5 | -3.00E-02 | 2026 | 6 | . |
| Singh et al, 2022 | 713 | Mental | TST | Cross-sectional | Asia | Well-being | . | Q-LES-Q-SF | 51 | -1.54E-01 | 2022 | 7 | 20.7 |
| Sirtoli et al, 2024 | 3,161 | Mental | TSM | Cross-sectional | South America | Depression | . | PHQ | 68.6 | 1.50E-01 | 2024 | 9 | 21.9 |
| Sy et al, 2024 | 1,001 | Physical | TST | Cross-sectional | North America | . | Sleep quality | PSQI | 71.1 | 1.20E-01 | 2024 | 9 | 19.9 |
| Thomson et al, 2008 | 613 | Physical | RST | Cross-sectional | North America | . | Weight | BMI | 64 | 0.18 | 2008 | 6 | . |
| Uwimana et al, 2022 | 452 | Physical | TST | Cross-sectional | Asia | . | Eye health | DEQ-5 | 34.1 | 9.20E-02 | 2022 | 6 | 27.25 |
| Walsh et al, 2020 | 2,390 | Physical | TST | Cross-sectional | North America | . | Sleep quality | The insomnia severity index | 73.4 | 0.186764486 | 2020 | 7 | 22.17 |
| Wang et al, 2022 | 9,716 | Mental | Smartphone | Cross-sectional | Asia | Depression | . | GAD-7 | 43.8 | 1.32E-01 | 2022 | 8 | . |
| Wang et al, 2023 | 1,070 | Mental | TST | Cross-sectional | Asia | Depression | . | CES-D | 56.9 | 3.07E-02 | 2023 | 7 | . |
| Watanabe et al, 2022 | 3,869 | Physical | TSM | Longitudinal | Asia | . | Sleep quality | Sleep problems were assessed based on the following question: “Do you have difficulty in sleeping?” Two response options were provided: “yes” and “no” | 35.8 | 1.54E-01 | 2022 | 8 | 22.7 |
| Wen et al, 2023 | 1,616 | Mental | TST | Cross-sectional | Asia | Depression | . | SAS | 54.6 | 1.40E-01 | 2023 | 5 |  |
| Wu et al, 2015 | 4,747 | Mental | TST | Cross-sectional | Asia | Depression | . | SAS | 58.4 | 1.69E-01 | 2015 | 8 | 19.22 |
| Wu et al, 2016 | 2,521 | Mental | RST | Longitudinal | Asia | Depression | . | CES-D | 52.9 | 1.85E-01 | 2016 | 8 | 18.53 |
| Xia et al, 2023 | 1,016 | Mental | Computer | Cross-sectional | Asia | Combined （Anxiety + Depression） | . | GAD-7 /PHQ-9 | 60.9 | 1.14E-01 | 2023 | 5 | . |
| Xia et al, 2025 | 3,190 | Mental | RST | Cross-sectional | Asia | Depression | . | CESD-10 | 32.8 | 4.55E-01 | 2025 | 7 | 20.2 |
| Xiao et al, 2025 | 1,160 | Mental | TST | Cross-sectional | Asia | Depression | . | CES-D | 59.7 | 4.42E-01 | 2025 | 6 | 20.2 |
| Yang et al, 2022 | 686 | Mental | Computer | Longitudinal | Asia | Combined （Anxiety + Depression + Stress） | . | DASS-21 | 0 | 2.10E-01 | 2022 | 5 | 21 |
| Zarban et al, 2024 | 402 | Physical | TST | Cross-sectional | Others | . | Eye health | OSDI | 72.9 | 3.45E-02 | 2024 | 4 | . |
| Zeleke et al, 2022 | 404 | Physical | TST | Cross-sectional | North America | . | Eye health | OSDI | 24.8 | 2.52E-01 | 2022 | 6 | 29 |
| Zhang et al, 2021 | 11,787 | Mental | TSM | Cross-sectional | Asia | Depression | . | PHQ-9 | 42.9 | 0.48 | 2021 | 8 | 20.51 |
| Zhang et al, 2025 | 2,896 | Mental | TST | Cross-sectional | Asia | Depression | . | PHQ-9 | 55.5 | 2.01E-01 | 2025 | 8 | 19.54 |
| Zhang et al, 2026 | 1,057 | Physical | RST | Longitudinal | Asia | . | Sleep quality | PSQI | 56.67 | 1.67E-01 | 2026 | 9 | 17.93 |
| Zhou et al, 2021 | 584 | Mental | RST | Cross-sectional | Asia | Depression | . | PHQ-9 | 40.2 | 1.18E-01 | 2021 | 7 | . |
| Zou et al, 2025 | 7,401 | Mental | Smartphone | Cross-sectional | Asia | Depression | . | SDS | 67.79 | 1.55E-01 | 2025 | 8 | . |
| Abbreviations:  PSQI: Pittsburgh Sleep Quality Index  PHQ-9: 9-item Patient Health Questionnaire  OSDI: Ocular Surface Disease Index  CVS-Q: Computer Vision Syndrome Questionnaire  DASS-21: 21-item Depression Anxiety Stress Scales  ASSQ: Athlete Sleep Screening Questionnaire  BMI: Body Mass Index  WHOQOL-BREF: World Health Organization Quality of Life-Brief Version  BaSIQS: The Basic Scale on Insomnia Symptoms and Quality of sleep  CES-D: Center for Epidemiological Studies Depression Sclae  SAS: Self-Rating Anxiety Scale  SDS: Self-Rating Depression Scale  SHMS V1.0: Sub-Health Measurement Scale Version 1.0  GAD-7: 7-item Generalized Anxiety Disorder Scale  CES-D10: 10-item Generalized Anxiety Disorder Scale  Q-LES-Q-SF: Quality of Life Enjoyment and Satisfaction Questionnaire-Short Form | | | | | | | | | | | | | |

**Table S4. Measurement characteristics of studies included in the eye health outcome category**

| **Study** | **Eye health construct** | **Measurement instrument** | **Validated instrument** | **Instrument used in standard form** | **Objective ophthalmological assessment** | **Notes on measurement** |
| --- | --- | --- | --- | --- | --- | --- |
| Alashqar et al., 2025 | DED | OSDI | Yes | Yes | No | Validated self-reported symptom questionnaire |
| Aljaberi et al., 2025 | DED | OSDI | Yes | Yes | No | Both instruments were reported as validated; symptom-based assessment only |
| Allwihan et al., 2024 | DED | CVS-Q | Yes | No | No | Several original symptom items were omitted; measurement concern noted |
| Alqurashi et al., 2024 | DED | OSDI | Yes | Yes | No | Standard OSDI scoring and severity classification used |
| Carter et al., 2021 | DED | DEQ-5 | Yes | Yes | No | Established validated instrument |
| Daniyal et al., 2022 | DES | Eye strain items from semi-structured questionnaire | No | No | No | No identifiable standard validated eye-health instrument |
| Ezinne et al., 2023 | SDED | OSDI | Yes | Yes | No | Standard 12-item OSDI used; additional risk-factor questions were collected separately. |
| Maqbool et al., 2024 | DES | CVS-Q | Yes | Yes | No | Standard validated CVS-Q used to assess symptom burden |
| Jakhar et al., 2023 | DES | content-validated structured online survey | No | No | No | Content validated, but not an established standardized DES/DED instrument |
| Rana et al., 2023 | DES | CVS-Q | Yes | Yes | No | Standard CVS-Q used; internal consistency in the study was high |
| Patni et al., 2023 | DED/DES | Self-developed item symptoms score | No | No | No | Using binary (yes/no) options, higher scores indicating greater symptom burden. |
| Zarban et al., 2024 | DED | Arabic OSDI | Yes | Yes | No | Validated Arabic OSDI used |
| Zeleke et al., 2022 | SDED | OSDI | Yes | Yes | No | Standard 12-item OSDI used; authors noted the lack of objective assessment as a limitation. |
| Uwimana et al., 2022 | DED | DEQ-5 | Yes | Yes | No | DED was assessed using DEQ-5 and DES using CVS-Q; both were validated symptom-based instruments. |
| Kumari et al., 2026 | DES | CVS-Q | Yes | Yes | No | Standard validated CVS-Q used; however, no formal population-specific linguistic/cultural validation was reported. |
| Abbreviations:  CVS-Q: computer vision syndrome questionnaire;  DED: dry eye disease  DEQ-5: 5-item dry eye questionnaire  DES: digital eye strain  OSDI: ocular surface disease index  Note:  The eye health outcome category primarily comprised symptom-based measures of dry eye disease and digital eye strain/computer vision syndrome. Most studies used established validated instruments, although one study used an adapted version of a previously validated questionnaire. Across studies, outcome assessment was predominantly self-reported and was generally not corroborated by objective ophthalmological examination. | | | | | | |
