# Supplementary material for: Association of Screen Time With Physical and Mental Health in University Students: Systematic Review and Meta-Analysis
Source: J Med Internet Res. 2026 Oct 26;28:e93848. doi: 10.2196/93848 (PMC13749025; doi:10.2196/93848)
Supplement: Supplementary file 6 [file jmir_v28i1e93848_app6.docx]

# **Table S5. Study Quality Evaluation Coding Criteria**

| Item | Description | Coding |
| --- | --- | --- |
| 1. Data source (DS) | Define the source of information (survey, record review). | 0=No 1=Yes |
| 2. Eligibility criteria (EC) | List inclusion and exclusion criteria for exposed and unexposed subjects (case and controls) or refer to previous publications. | 0=No 1=Yes |
| 3. Study period (SP) | Indicate time periods used for identifying patients. | 0=No 1=Yes |
| 4. Sampling method (SM) | Indicate whether or not subjects were consecutive if not population based. | 0=No 1=Yes |
| 5. Blinding (BL) | Indicate if evaluators of subjective components of study were masked to other aspects of the status of the participants. | 0=No 1=Yes |
| 6. Quality control (QC) | Describe any assessments undertaken for quality assurance purposes (eg, test/retest of primary outcomes measurements). | 0=No 1=Yes |
| 7. Exclusions (EX) | Explain any patient exclusions from analysis. | 0=No 1=Yes |
| 8. Confounding control (CC) | Describe how confounding was assessed and/or controlled. | 0=No 1=Yes |
| 9. Missing data (MD) | If applicable, explain how missing data was handled in the analysis. | 0=No 1=Yes |
| 10. Response rate (RR) | Summarize patient response rates and complete data collection. | 0=No 1=Yes |
| 11. Follow-up (FU) | Clarify what follow-up, if any, was expected and the percentage of patients for which incomplete data or follow-up was obtained. | 0=No 1=Yes |

# **Table S6. Study Quality Scoring for Each Study Included in the Meta-analysis**

| **Study** | **DS** | **EC** | **SP** | **SM** | **BL** | **QC** | **EX** | **CC** | **MD** | **RR** | **FU** | **Scores** | **Quality** |
| --- | --- | --- | --- | --- | --- | --- | --- | --- | --- | --- | --- | --- | --- |
| Achak et al, 2023 | 1 | 0 | 1 | 1 | 0 | 1 | 0 | 0 | 0 | 0 | 1 | 5 | Moderate quality |
| Akca et al, 2019 | 1 | 0 | 0 | 1 | 0 | 0 | 0 | 0 | 0 | 0 | 1 | 3 | Low quality |
| Akçay D & Akçay B, 2020 | 1 | 1 | 1 | 0 | 0 | 0 | 1 | 1 | 1 | 0 | 1 | 7 | Moderate quality |
| Akcay et al, 2020 | 1 | 1 | 1 | 0 | 0 | 0 | 1 | 1 | 0 | 0 | 1 | 6 | Moderate quality |
| Akowuah et al, 2021 | 1 | 1 | 1 | 1 | 0 | 0 | 1 | 1 | 1 | 0 | 1 | 8 | High quality |
| Al-Amin et al, 2025 | 1 | 1 | 1 | 0 | 0 | 0 | 1 | 1 | 1 | 0 | 0 | 6 | Moderate quality |
| Alashqar et al, 2025 | 1 | 1 | 1 | 0 | 0 | 0 | 1 | 1 | 0 | 1 | 0 | 6 | Moderate quality |
| Aljaberi et al, 2025 | 1 | 0 | 1 | 0 | 0 | 0 | 0 | 1 | 0 | 1 | 0 | 4 | Moderate quality |
| Allwihan et al, 2024 | 1 | 1 | 1 | 1 | 0 | 0 | 1 | 1 | 0 | 1 | 0 | 7 | Moderate quality |
| Almalki et al, 2026 | 1 | 1 | 1 | 1 | 0 | 0 | 0 | 1 | 0 | 0 | 0 | 5 | Moderate quality |
| Almeida et al, 2022 | 1 | 0 | 1 | 0 | 0 | 0 | 0 | 1 | 1 | 0 | 1 | 5 | Moderate quality |
| Alqurashi et al, 2024 | 1 | 1 | 1 | 1 | 0 | 0 | 0 | 0 | 0 | 0 | 0 | 4 | Moderate quality |
| Amez et al, 2020 | 1 | 0 | 1 | 0 | 0 | 0 | 1 | 1 | 1 | 0 | 0 | 5 | Moderate quality |
| Anuradha et al, 2022 | 1 | 1 | 1 | 1 | 0 | 0 | 1 | 0 | 0 | 1 | 0 | 6 | Moderate quality |
| Aridi et al, 2026 | 1 | 1 | 1 | 1 | 0 | 0 | 0 | 0 | 0 | 0 | 0 | 4 | Moderate quality |
| Aschale et al, 2024 | 1 | 1 | 1 | 1 | 0 | 1 | 0 | 1 | 1 | 1 | 1 | 9 | High quality |
| Balpande et al, 2024 | 1 | 1 | 1 | 1 | 0 | 0 | 0 | 1 | 0 | 1 | 1 | 7 | Moderate quality |
| Barry et al, 2022 | 1 | 0 | 1 | 1 | 0 | 0 | 0 | 0 | 0 | 1 | 1 | 5 | Moderate quality |
| Benaich et al, 2021 | 1 | 1 | 1 | 1 | 0 | 0 | 0 | 0 | 0 | 1 | 0 | 5 | Moderate quality |
| Brar et al, 2026 | 1 | 0 | 1 | 1 | 0 | 0 | 0 | 1 | 0 | 1 | 1 | 6 | Moderate quality |
| Brown et al, 2022 | 1 | 0 | 1 | 1 | 0 | 0 | 1 | 1 | 1 | 1 | 1 | 8 | High quality |
| Cartes et al, 2021 | 1 | 0 | 1 | 0 | 0 | 0 | 1 | 0 | 0 | 1 | 1 | 5 | Moderate quality |
| Chandler et al, 2024 | 1 | 0 | 1 | 0 | 0 | 0 | 1 | 1 | 1 | 0 | 1 | 6 | Moderate quality |
| Chen et al, 2024 | 1 | 1 | 1 | 0 | 0 | 0 | 1 | 1 | 1 | 1 | 1 | 8 | High quality |
| Daniyal et al, 2022 | 1 | 1 | 1 | 1 | 0 | 0 | 0 | 0 | 0 | 0 | 1 | 5 | Moderate quality |
| Deyo et al, 2024 | 1 | 1 | 1 | 0 | 0 | 0 | 0 | 1 | 0 | 0 | 1 | 5 | Moderate quality |
| Ezinne et al, 2023 | 1 | 1 | 1 | 1 | 0 | 0 | 0 | 1 | 0 | 1 | 1 | 7 | Moderate quality |
| Fang et al, 2023 | 1 | 0 | 1 | 0 | 0 | 0 | 0 | 0 | 1 | 0 | 1 | 4 | Moderate quality |
| Feng et al, 2014 | 1 | 1 | 1 | 1 | 0 | 0 | 0 | 1 | 1 | 1 | 1 | 8 | High quality |
| Galle et al, 2025 | 1 | 0 | 1 | 1 | 0 | 0 | 0 | 1 | 0 | 0 | 0 | 4 | Moderate quality |
| Garcia et al, 2024 | 1 | 1 | 1 | 1 | 0 | 0 | 1 | 1 | 0 | 0 | 0 | 6 | Moderate quality |
| Ge et al, 2020 | 1 | 0 | 1 | 0 | 0 | 0 | 0 | 1 | 0 | 0 | 0 | 3 | Low quality |
| Hjetland et al, 2021 | 1 | 1 | 1 | 1 | 0 | 0 | 1 | 1 | 1 | 1 | 1 | 9 | High quality |
| Hosen et al, 2021 | 1 | 0 | 1 | 1 | 0 | 0 | 1 | 1 | 0 | 1 | 1 | 7 | Moderate quality |
| Huang et al, 2020 | 1 | 1 | 1 | 0 | 0 | 0 | 1 | 0 | 1 | 0 | 1 | 6 | Moderate quality |
| Huang et al, 2022 | 1 | 0 | 0 | 0 | 0 | 0 | 0 | 0 | 0 | 0 | 1 | 2 | Low quality |
| Jakhar et al, 2023 | 1 | 0 | 0 | 0 | 0 | 0 | 1 | 0 | 1 | 0 | 1 | 4 | Moderate quality |
| Kaewpradit et al, 2025 | 1 | 1 | 1 | 1 | 0 | 0 | 1 | 1 | 1 | 1 | 0 | 8 | High quality |
| Kalirathinam et al, 2019 | 1 | 1 | 0 | 0 | 0 | 0 | 0 | 0 | 0 | 0 | 0 | 2 | Low quality |
| Kazhali et al, 2021 | 1 | 1 | 1 | 1 | 0 | 0 | 0 | 0 | 0 | 0 | 1 | 5 | Moderate quality |
| Koly et al, 2021 | 1 | 0 | 1 | 1 | 0 | 0 | 0 | 1 | 0 | 1 | 0 | 5 | Moderate quality |
| Krishnan et al, 2020 | 1 | 1 | 1 | 0 | 0 | 0 | 1 | 0 | 1 | 0 | 1 | 6 | Moderate quality |
| Kumari et al, 2026 | 1 | 1 | 1 | 1 | 0 | 0 | 1 | 1 | 1 | 0 | 0 | 7 | Moderate quality |
| Lavados-Romo et al, 2021 | 1 | 1 | 1 | 1 | 0 | 0 | 1 | 1 | 1 | 1 | 1 | 9 | High quality |
| Leung et al, 2021 | 1 | 1 | 0 | 0 | 0 | 0 | 0 | 1 | 0 | 0 | 1 | 4 | Moderate quality |
| Liebig et al, 2023 | 1 | 1 | 1 | 1 | 0 | 0 | 1 | 1 | 0 | 1 | 0 | 7 | Moderate quality |
| Liu et al, 2023 | 1 | 1 | 1 | 0 | 0 | 0 | 1 | 0 | 0 | 0 | 1 | 5 | Moderate quality |
| Lu et al, 2023 | 1 | 1 | 1 | 1 | 0 | 0 | 1 | 1 | 0 | 1 | 1 | 8 | High quality |
| Ma et al, 2020 | 1 | 0 | 1 | 0 | 0 | 0 | 1 | 1 | 0 | 1 | 1 | 6 | Moderate quality |
| Maqbool et al, 2024 | 1 | 1 | 1 | 0 | 0 | 0 | 0 | 0 | 0 | 0 | 0 | 3 | Low quality |
| Meng et al, 2021 | 1 | 0 | 1 | 0 | 0 | 0 | 0 | 1 | 1 | 1 | 1 | 6 | Moderate quality |
| Noel et al, 2022 | 1 | 1 | 1 | 0 | 0 | 0 | 1 | 1 | 0 | 0 | 1 | 6 | Moderate quality |
| Patni et al, 2026 | 1 | 1 | 1 | 1 | 0 | 0 | 0 | 1 | 0 | 0 | 0 | 5 | Moderate quality |
| Peltzer et al, 2014 | 1 | 0 | 0 | 1 | 0 | 0 | 0 | 1 | 0 | 1 | 1 | 5 | Moderate quality |
| Pham et al, 2021 | 1 | 1 | 1 | 0 | 0 | 0 | 1 | 1 | 0 | 0 | 1 | 6 | Moderate quality |
| Rakicioglu N et al, 2026 | 1 | 1 | 0 | 1 | 0 | 0 | 0 | 0 | 0 | 0 | 0 | 3 | Low quality |
| Rana et al, 2023 | 1 | 1 | 1 | 0 | 0 | 0 | 0 | 0 | 0 | 0 | 1 | 4 | Moderate quality |
| Rosenthal et al, 2021 | 1 | 0 | 1 | 0 | 0 | 0 | 1 | 1 | 0 | 0 | 1 | 5 | Moderate quality |
| Sarker et al, 2026 | 1 | 0 | 0 | 0 | 0 | 1 | 1 | 1 | 0 | 0 | 0 | 4 | Moderate quality |
| Singh et al, 2022 | 1 | 1 | 1 | 1 | 0 | 0 | 1 | 0 | 0 | 0 | 0 | 5 | Moderate quality |
| Sirtoli et al, 2024 | 1 | 1 | 1 | 1 | 0 | 0 | 1 | 1 | 1 | 1 | 1 | 9 | High quality |
| Sy et al, 2024 | 1 | 1 | 1 | 1 | 0 | 0 | 1 | 1 | 1 | 0 | 0 | 7 | Moderate quality |
| Thomson et al, 2008 | 1 | 1 | 1 | 1 | 0 | 0 | 0 | 0 | 0 | 1 | 1 | 6 | Moderate quality |
| Uwimana et al, 2022 | 1 | 0 | 0 | 0 | 0 | 0 | 1 | 1 | 1 | 0 | 1 | 5 | Moderate quality |
| Walsh et al, 2020 | 1 | 1 | 1 | 0 | 0 | 0 | 1 | 1 | 1 | 0 | 1 | 7 | Moderate quality |
| Walsh et al, 2020 | 1 | 1 | 1 | 0 | 0 | 0 | 1 | 1 | 1 | 0 | 1 | 7 | Moderate quality |
| Wang et al, 2022 | 1 | 1 | 1 | 0 | 0 | 0 | 1 | 1 | 1 | 1 | 1 | 8 | High quality |
| Wang et al, 2023 | 1 | 1 | 1 | 0 | 0 | 0 | 1 | 1 | 0 | 1 | 1 | 7 | Moderate quality |
| Watanabe et al, 2022 | 1 | 1 | 1 | 1 | 0 | 0 | 1 | 1 | 0 | 1 | 1 | 8 | High quality |
| Wen et al, 2023 | 1 | 0 | 0 | 0 | 0 | 1 | 1 | 1 | 0 | 0 | 1 | 5 | Moderate quality |
| Wu et al, 2015 | 1 | 1 | 1 | 1 | 0 | 0 | 0 | 1 | 0 | 1 | 0 | 6 | Moderate quality |
| Wu et al, 2016 | 1 | 1 | 1 | 1 | 0 | 0 | 1 | 1 | 0 | 1 | 1 | 8 | High quality |
| Xia et al, 2023 | 1 | 0 | 1 | 1 | 0 | 0 | 0 | 0 | 0 | 1 | 1 | 5 | Moderate quality |
| Xia et al, 2025 | 1 | 0 | 0 | 1 | 0 | 0 | 1 | 1 | 0 | 0 | 0 | 4 | Moderate quality |
| Xiao et al, 2025 | 1 | 0 | 0 | 1 | 0 | 0 | 0 | 1 | 1 | 1 | 0 | 5 | Moderate quality |
| Yang et al, 2021 | 1 | 1 | 1 | 1 | 0 | 0 | 1 | 1 | 0 | 0 | 1 | 7 | Moderate quality |
| Zarban et al, 2024 | 1 | 0 | 1 | 0 | 0 | 0 | 0 | 0 | 0 | 1 | 1 | 4 | Moderate quality |
| Zeleke et al, 2022 | 1 | 1 | 1 | 1 | 0 | 0 | 0 | 0 | 0 | 1 | 1 | 6 | Moderate quality |
| Zhang et al, 2022 | 1 | 1 | 1 | 1 | 0 | 0 | 1 | 1 | 1 | 1 | 0 | 8 | High quality |
| Zhang et al, 2025 | 1 | 1 | 1 | 1 | 0 | 0 | 1 | 1 | 0 | 0 | 0 | 6 | Moderate quality |
| Zhang et al, 2026 | 1 | 1 | 1 | 1 | 0 | 0 | 1 | 1 | 1 | 0 | 1 | 8 | High quality |
| Zhou et al, 2021 | 1 | 1 | 1 | 0 | 0 | 0 | 1 | 1 | 0 | 1 | 0 | 6 | Moderate quality |
| Zou et al, 2025 | 1 | 1 | 1 | 1 | 0 | 1 | 1 | 1 | 1 | 0 | 0 | 8 | High quality |
