# Supplementary material for: Association of Screen Time With Physical and Mental Health in University Students: Systematic Review and Meta-Analysis
Source: J Med Internet Res. 2026 Oct 26;28:e93848. doi: 10.2196/93848 (PMC13749025; doi:10.2196/93848)
Supplement: Supplementary file 7 [file jmir_v28i1e93848_app7.docx]

**Table S7. GRADE Certainty of Evidence for the Association Between Screen Time and Health Outcomes**

| **Outcome** | **Samples (k)** | **Sample size (N)** | **Pooled r (95% CI)** | **Risk of bias** | **Inconsistency** | **Indirectness** | **Imprecision** | **Certainty** |
| --- | --- | --- | --- | --- | --- | --- | --- | --- |
| Depression | 21 | 54,620 | 0.230 (0.167-0.291) | Not serious | Serious ^a^ | Not serious | Not serious | Very low |
| Combined mental health | 8 | 7,435 | 0.170 (0.076-0.261) | Serious ^b^ | Serious ^c^ | Serious ^d^ | Not serious | Very low |
| Well-being | 7 | 12,163 | 0.040 (-0.146 to 0.223) | Not serious | Very serious ^e^ | Not serious | Serious ^f^ | Very low |
| Stress | 2 | 1,137 | 0.103 (-0.222 to 0.407) | Serious ^g^ | Not serious | Not serious | Very serious ^h^ | Very low |
| Sleep quality | 26 | 75,213 | 0.120 (0.080-0.157) | Not serious | Serious ^i^ | Not serious | Not serious | Very low |
| Eye health | 16 | 9,331 | 0.277 (0.089-0.446) | Serious ^j^ | Very serious ^k^ | Not serious | Serious ^l^ | Very low |
| Weight | 3 | 1,441 | 0.071( -0.222 to 0.351) | Serious ^m^ | Serious ^n^ | Not serious | Very serious ^o^ | Very low |
| ^a^: Downgraded 1 level for considerable heterogeneity (I^2^ =97.53%, τ²=0.019).  ^b^: Downgraded 1 level because 7 of 8 contributing samples were of moderate methodological quality, with only 1 high-quality sample.  ^c^: Downgraded 1 level for considerable heterogeneity (I^2^ =89.10%, τ²=0.011).  ^d^: Downgraded 1 level because the composite category included 2 or more mental health constructs that varied across studies.  ^e^: Downgraded 2 levels for extreme between-study heterogeneity (I^2^ =97.92%, τ²=0.040).  ^f^: Downgraded 1 level because the 95% CI crossed the null and both negative and positive associations of potentially meaningful magnitude (95% CI -0.146 to 0.223).  ^g^: Downgraded 1 level for risk of bias because both contributing samples were of low methodological quality  ^h:^ Downgraded 2 levels for very serious imprecision because only 2 samples contributed to the estimate and the 95% CI was very wide, spanning negative, null, and positive associations (95% CI -0.222 to 0.407).  ^I:^ Downgraded 1 level for considerable between-study heterogeneity (I²=93.50%, τ²=0.007).  ^J:^ Downgraded 1 level for risk of bias because 15 of 16 contributing samples were of moderate methodological quality and 1 was of low methodological quality, with no high-quality evidence.  ^K:^ Downgraded 2 levels for extreme between-study heterogeneity (I²=98.09%, τ²=0.123).  ^L:^ Downgraded 1 level for imprecision because the 95% CI was wide and encompassed associations ranging from relatively small to substantially larger magnitudes (95% CI 0.089 to 0.446).  ^M:^ Downgraded 1 level for risk of bias because 1 of 3 contributing samples was of low methodological quality and the remaining 2 were of moderate quality, with no high-quality evidence.  ^N:^ Downgraded 1 level for considerable between-study heterogeneity (I²=77.34%, τ²=0.010).  **^O:^** Downgraded 2 levels for very serious imprecision because only 3 studies contributed to the estimate and the 95% CI was very wide, spanning negative, null, and positive associations (95% CI -0.222 to 0.351).  **Abbreviations:** CI, confidence interval; GRADE, Grading of Recommendations Assessment, Development and Evaluation.  **Note:** k indicates the number of independent analytic samples. | | | | | | | |  |
