# Supplementary material for: Association of Screen Time With Physical and Mental Health in University Students: Systematic Review and Meta-Analysis
Source: J Med Internet Res. 2026 Oct 26;28:e93848. doi: 10.2196/93848 (PMC13749025; doi:10.2196/93848)
Supplement: Supplementary file 8 [file jmir_v28i1e93848_app8.docx]

Figure S1. Funnel plot assessing small sample study effect in the overall association between screen time and health outcomes among university students. Each square represents an included sample. The vertical dotted line indicates the pooled correlation coefficient, and the diagonal dotted lines represent the pseudo 95% confidence limits.
