# Supplementary material for: Association of Screen Time With Physical and Mental Health in University Students: Systematic Review and Meta-Analysis
Source: J Med Internet Res. 2026 Oct 26;28:e93848. doi: 10.2196/93848 (PMC13749025; doi:10.2196/93848)
Supplement: Supplementary file 9 [file jmir_v28i1e93848_app9.docx]

Figure S2. Leave-one-out sensitivity analysis of the association between screen time and health outcomes. Each point represents the pooled Pearson correlation coefficient (*r*) after omission of the indicated effect size, with horizontal lines representing 95% CIs. The vertical dashed line indicates the pooled estimate from the primary REML-HKSJ analysis (*r*=0.175). The pooled estimates ranged from 0.164 to 0.180 and remained statistically significant after omission of any single effect size.

Figure S3: Influence diagnostics for the overall random-effects meta-analysis of the association between screen time and health outcomes among university students. Diagnostic measures include externally standardized residuals, DFFITS, Cook distance, covariance ratios, τ² and Q statistics with each study deleted, hat values, and model weights. Diamond symbols highlight studies 15 [1] and 44 [2]as potentially influential observations. Dashed horizontal lines indicate conventional diagnostic thresholds where applicable. Q: Cochran Q statistic; τ²: between-study variance

| **Table S8. Sensitivity analyses of the associations between screen time and eye health outcomes** | | | | | | |  |
| --- | --- | --- | --- | --- | --- | --- | --- |
| Analysis | k | r | 95% CI | P | I^2^ (%) | Τ^2^ | |
| Primary eye-health analysis | 16 | 0.277 | 0.089-0.446 | .007 | 98.09 | 0.123 | |
| Restricted to established validated instruments used in standard form | 12 | 0.288 | 0.038-0.503 | .03 | 98.54 | 0.152 | |

Reference

1.	Aridi M, Alwan N, Ghach W. Behavioral and cultural determinants of symptomatic dry eye disease among university students in the UAE. Plos One. 2026 Feb 26;21(2). PMID: WOS:001702745100035. doi: 10.1371/journal.pone.0343508.

2.	Kumari S, Maran YM, Krishnan KA, Raja M, S G, Paraman V. Digital eye strain among Indian university students in the post-COVID era: a cross-sectional study. Front Public Health. 2026;14:1829471. PMID: 42338538. doi: 10.3389/fpubh.2026.1829471.
